# Antimicrobial peptides do not directly contribute to aging in *Drosophila*, but improve lifespan by preventing dysbiosis

**DOI:** 10.1242/dmm.049965

**Published:** 2023-04-26

**Authors:** Mark A. Hanson, Bruno Lemaitre

**Affiliations:** Global Health Institute, School of Life Science, École Polytechnique Fédérale de Lausanne (EPFL), 1015 Lausanne, Switzerland

**Keywords:** NF-κB, Aging, Antimicrobial peptide, *Drosophila*, Host defence peptide, Inflammation

## Abstract

Antimicrobial peptides (AMPs) are innate immune effectors first studied for their role in host defence. Recent studies have implicated these peptides in the clearance of aberrant cells and in neurodegenerative syndromes. In *Drosophila*, many AMPs are produced downstream of Toll and Imd NF-κB pathways upon infection. Upon aging, AMPs are upregulated, drawing attention to these molecules as possible causes of age-associated inflammatory diseases. However, functional studies overexpressing or silencing these genes have been inconclusive. Using an isogenic set of AMP gene deletions, we investigated the net impact of AMPs on aging. Overall, we found no major effect of individual AMPs on lifespan, with the possible exception of Defensin. However, *ΔAMP14* flies lacking seven AMP gene families displayed reduced lifespan. Increased bacterial load in the food of aged *ΔAMP14* flies suggested that their lifespan reduction was due to microbiome dysbiosis, consistent with a previous study. Moreover, germ-free conditions extended the lifespan of *ΔAMP14* flies. Overall, our results did not point to an overt role of individual AMPs in lifespan. Instead, we found that AMPs collectively impact lifespan by preventing dysbiosis during aging.

## Teaser

**Summary:** Antimicrobial peptides are important immune molecules to fight microbes that are upregulated upon aging. Our results suggest that their upregulation upon aging is likely a consequence of aging, rather than a cause.

## INTRODUCTION

Antimicrobial peptides (AMPs) are innate immune effectors found in plants and animals that show microbicidal activity *in vitro*. They are typically cationic and amphipathic, and disrupt microbial membranes that are more negatively charged (Broekaert et al., 1997; Christensen et al., 1988; Ludtke et al., 1996; Zasloff, 1987). Recent studies in various animal models have pointed to a role for AMPs beyond microbial infection, notably in aging and aging-related diseases (Deslouches and Di, 2017; Mookherjee et al., 2020; Semple and Dorin, 2012; Smith and Gwyer Findlay, 2022). Like other animals, genes encoding AMPs (hereafter AMP genes) are upregulated upon aging in *Drosophila*, and disparate studies have suggested a role for AMPs as causative agents of aging, neurodegeneration, or mitochondrial stress (Arora and Ligoxygakis, 2020; Garschall and Flatt, 2018; Hanson and Lemaitre, 2020). Here, we have leveraged a set of isogenic AMP-deficient flies to analyse the contribution of AMPs to aging in *Drosophila melanogaster*.

AMPs are well characterized in *Drosophila* for their contribution to host defence. There are currently eight families of inducible AMPs known in *D. melanogaster*: the antifungals Drosomycin (Drs), Baramicin (Bara) and Metchnikowin (Mtk); Cecropins (Cec) and Defensin (Def), which have both antibacterial and some antifungal activities; and Drosocin (Dro), Attacins (Att) and Diptericins (Dpt), which primarily exhibit antibacterial activity (Hanson et al., 2021; Imler and Bulet, 2005). In addition, the *Drosophila* genome encodes many other host defence peptide families such as Daisho (Dso) and Bomanin (Bom), for which overt antimicrobial activity *in vitro* has not yet been demonstrated, but functional studies have shown that they are important *in vivo* to resist microbial infection (Clemmons et al., 2015; Cohen et al., 2020). Although most *Drosophila* defence-peptide-encoding genes are strongly induced in the fat body in response to systemic infections, many show specific and complex patterns of expression in tissues such as the trachea, gut, salivary glands or reproductive tracts (Ferrandon et al., 1998; Tzou et al., 2000).

AMP genes are regulated at the transcriptional level by the Toll and Imd NF-κB signalling pathways upon systemic infection, or by the Imd pathway in local epithelia (Lemaitre and Hoffmann, 2007; Myllymäki et al., 2014). It is well established that Imd- and Toll-deficient flies show marked susceptibility to microbial infection. Until recently, the importance of immune effectors downstream of these pathways, notably AMPs, was unclear. With the advent of CRISPR gene editing, we systematically deleted seven families of AMP genes (Defensin, Cecropin, Drosocin, Attacin, Diptericin, Drosomycin and Metchnikowin) and analysed their contributions to host defence individually or collectively. We found that *Drosophila* AMPs are essential downstream of the Imd pathway to resist systemic infection by Gram-negative bacteria. *Drosophila* AMPs also contribute downstream of the Toll pathway to combat fungal and, to a lesser extent, Gram-positive bacterial infection, although Bomanins play a more prominent role against these micro-organisms (Carboni et al., 2021; Clemmons et al., 2015; Hanson et al., 2019a). Use of fly lines carrying combinations of AMP mutations revealed that they can function either additively or synergistically against some microbes, but, in some cases, AMPs exhibit striking specificity, with one peptide contributing most of the AMP-dependent defence against a specific pathogen (Hanson et al., 2019a, 2022a,b; Unckless et al., 2016). *Drosophila* AMPs are also important to control the fly microbiome downstream of the Imd pathway, particularly for their role in regulating Gram-negative bacteria like *Acetobacter* (Marra et al., 2021).

Although *Drosophila* AMPs were initially investigated for their contribution to host defence, AMP upregulation is also observed in non-immune contexts, including anti-tumour defence (Araki et al., 2019; Krautz et al., 2020; Parvy et al., 2019), neurodegeneration (Cao et al., 2013; Kounatidis et al., 2017; Petersen et al., 2013; Shukla et al., 2019; van Alphen et al., 2022) and aging (Glittenberg et al., 2011; Lai et al., 2007). This suggests that AMPs could have roles beyond their traditional function as microbicidal agents. Notably, transcriptomic studies revealed that high AMP expression, reflecting an increase in Imd pathway activity, is a hallmark of aging in flies (Landis et al., 2004; Pletcher et al., 2002; Seroude et al., 2002; Zerofsky et al., 2005). This is reminiscent of the situation observed in humans where a low-grade chronic inflammation is observed upon aging, termed ‘inflammaging’ (Franceschi et al., 2000, 2017; Liang and Diana, 2020). The key question now is to decipher whether this high and chronic activation of the immune response is simply a marker of aging, or whether cytotoxic immune effectors like AMPs accelerate aging-associated syndromes.

To address this question, several reports have investigated the role of the Imd pathway and AMPs in aging in *Drosophila* with contrasting outcomes. Most of these studies targeting Imd itself or the Imd transcription factor *Relish* (*Rel*) report that Imd pathway mutants are short-lived, (Cai et al., 2021; Kounatidis et al., 2017; Petersen et al., 2013). However, one study suggested that mutation of the *Imd* gene itself improved lifespan, as did fat-body-specific or whole-body AMP knockdown (Lin et al., 2018). The negative impact of Imd pathway downregulation has been associated with defects in gut homeostasis (Buchon et al., 2009), exaggerating the decline in gut resilience upon aging (Rera et al., 2012), leading to invasion of microbes into the hemolymph that drive mortality (Clark et al., 2015). Indeed, the guts of aged *Relish* mutant flies display precocious loss of compartmentalization, increased permeability and dysplasia (Liu et al., 2022). However, preventing the over-activation of Imd in the gut through transgene expression can improve lifespan, suggesting a careful balance of immune signalling in the gut is needed for optimal health with aging (Cai et al., 2021; Guo et al., 2014; Iatsenko et al., 2018). Some of these symptoms could be reverted when flies were raised axenically, pointing to a role of the microbiome in the precocious aging of the gut (Buchon et al., 2009; Clark et al., 2015; Iatsenko et al., 2018; Liu et al., 2022; Zhu et al., 2021). Another study reports that AMP genes are strongly induced in the head of old flies and that silencing *Relish* in glia can extend lifespan (Kounatidis et al., 2017). This would suggest that Imd-mediated immune responses drive aging by directly affecting brain activity. Indeed, many studies report that suppression of the Imd pathway can prevent neurodegeneration in various neurodegenerative disease models. For instance, flies mutated in the serine/threonine kinase *ataxia telangiectasia mutated* (*ATM*, also called *tefu*) display reduced lifespan associated with increased neuronal lesions, which can be rescued by combination with *Relish* mutation (Petersen et al., 2013). *Relish* deletion also rescues the aging-dependent neurodegeneration of *Cdk5α* mutant flies (Shukla et al., 2019), and interesting recent studies even showed that deletion of AMPs can improve fly survival after traumatic brain injury (Swanson et al., 2020; van Alphen et al., 2022). An effect of AMP overexpression in aging has also been proposed, albeit with conflicting results. Cao et al. (2013) reported AMP upregulation and neurodegeneration in the brains of *dnr1* mutant flies, and showed that AMP overexpression in neurons was sufficient to induce neurodegeneration. Badinloo et al. (2018) found that chronic and ubiquitous AMP overexpression reduced fly lifespan alongside induction of mitochondrial stress. A recent study of the AMP gene *Metchnikowin* further suggests a trade-off between greater antimicrobial activity and host fitness (Perlmutter et al., 2023). These results suggest AMPs could be deleterious to host fitness, which is also supported by evolutionary studies showing that AMP deletions segregate in wild populations (Early et al., 2017; Hanson et al., 2019b). However, in direct contradiction to this idea, an earlier report found that excess expression of certain AMPs could extend lifespan (Loch et al., 2017).

Presently, we do not know how AMPs impact aging, and a direct link between AMPs and aging remains to be demonstrated. Some of the conflicting results mentioned above may arise from use of different conditions (e.g. temperature, sex, mating status, nutrition) (Camus et al., 2019; Dobson et al., 2018; Landis et al., 2015; Miquel et al., 1976), microbiome differences, which could help explain contradictory findings on the impact of germ-free conditions on lifespan (Brummel et al., 2004; Ren et al., 2007), or lack of control over genetic background, including transgene insertions (Ferreiro et al., 2017; Sasaki et al., 2021), all of which can influence longevity. In the absence of mutants, these studies have relied on the use of over-expression or RNAi to modulate AMPs – methodologies with certain limitations (Tower et al., 2017; van der Graaf et al., 2022). As a result, exactly how AMPs contribute to aging remains unclear.

Here, we leveraged a recently generated collection of AMP mutations to analyse whether these immune effectors impact aging. We found that individual AMP deletions did not markedly affect aging, with the possible exception of *Defensin*. However, *ΔAMP14* flies lacking 14 AMP genes displayed reduced lifespan associated with microbial dysbiosis. Rearing *ΔAMP14* flies in germ-free conditions significantly rescued lifespan, indicating that AMPs contribute to lifespan through their impact on the microbiome. In contrast, depleting the microbiome of *Relish* mutant flies did not rescue lifespan, suggesting the Imd pathway can affect lifespan independent of its regulation of AMP genes. Together, our results indicate that AMPs are likely not direct contributors to the aging process, though they have a major impact on aging through AMP-microbiome interactions. Thus, our study, using loss-of-function mutations, clarifies the role of these innate immune effectors in aging.

## RESULTS

### The presence of cryptic infections may confound lifespan analyses in *Drosophila*

**Fig. 1.**
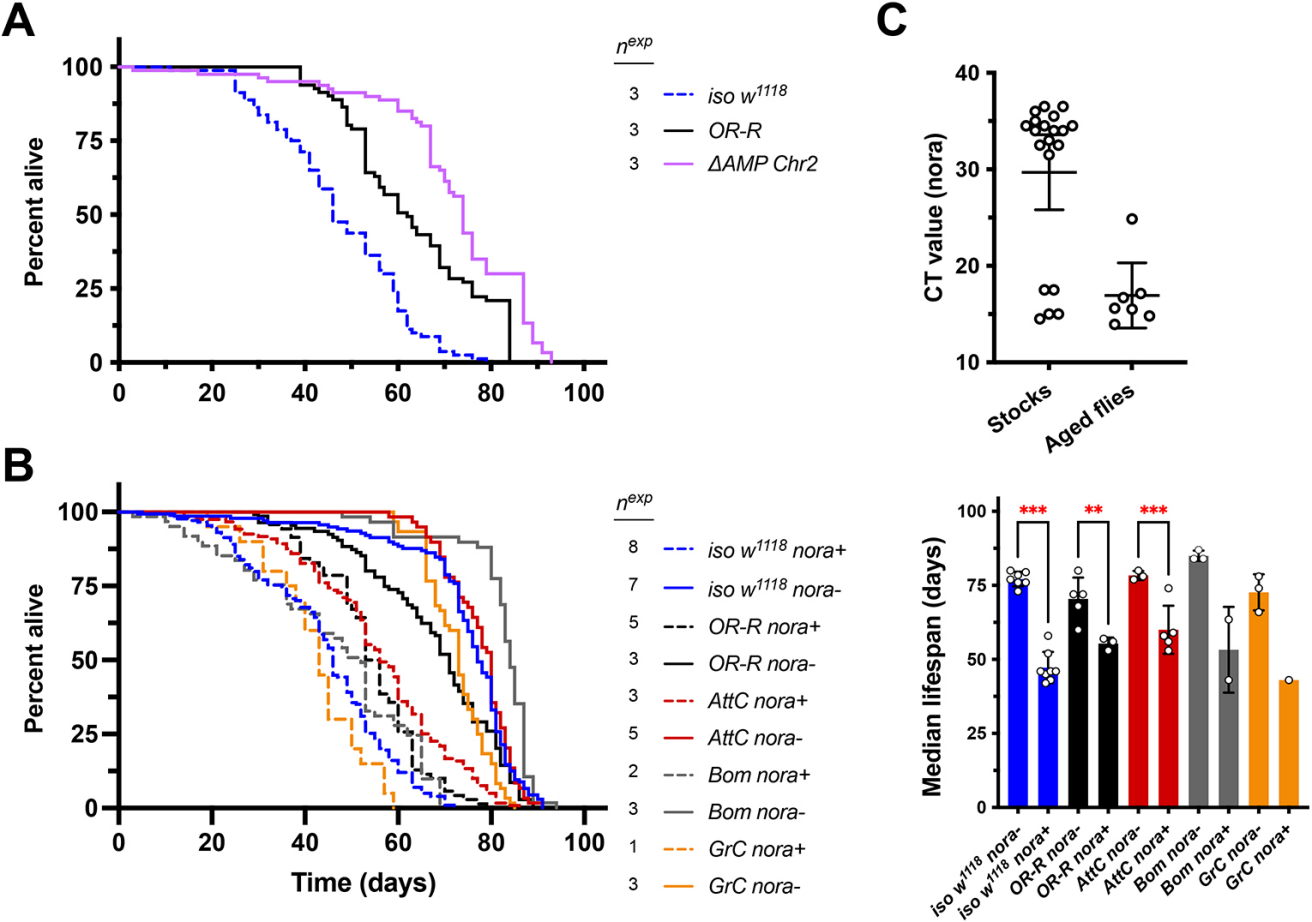
***Drosophila* Nora virus significantly reduces lifespan of DrosDel *iso w^1118^* flies.** Results for male flies are reported here and for female flies in Fig. S1. (A) Comparison of lifespan from early experiments using two wild-type strains (*iso w^1118^* and *OR-R*) alongside compound AMP mutants lacking *Def*, *Dro*, *AttA*, *AttB*, *AttC*, *Mtk*, *DptA* and *DptB*, which are deleted in *ΔAMP Chr2* flies. (B) Effect of Nora clearance on lifespan of *iso w^1118^*, *OR-R*, *AttC*, *Bom* and *Group C* (*GrC*) genotypes. Median lifespans are shown in the right panel (**P<0.01, ***P<0.001). Number of independent experiments (*n^exp^*) is reported. (C) Nora titres measured by cycle threshold (CT) values in 18°C source stocks (Stocks) or Nora-positive flies aged 3+ weeks kept at densities of 20 flies per vial (Aged flies). CT values represent Nora titre from 5 ng total fly RNA per 10 μl qPCR reaction. Bars show the mean±s.d.

To address the role of AMP genes in aging, we first compared the lifespan of flies lacking eight AMPs located on chromosome II (*ΔAMP Chr2* lacking *Def*, *Dro*, *AttA*, *AttB*, *AttC*, *Mtk*, *DptA* and *DptB*) to the lifespan of their DrosDel isogenic wild-type (*iso w^1118^*) controls. Of note, all our mutations are in the DrosDel isogenic genetic background (unless mentioned otherwise) and are negative for the endosymbiont *Wolbachia*. We show the lifespans of male flies in the main figures and those of female flies in the supplementary figures. In most cases, trends were similar between the two sexes. Cases where trends differed between males and females are noted in the main text. We observed a striking effect where *ΔAMP Chr2*-deficient flies displayed a marked lifespan extension compared to wild-types (Fig. 1A). This first result is consistent with studies suggesting that AMPs negatively impact lifespan. However, we were surprised by the short lifespan of our *iso w^1118^* wild-type flies compared to wild-type lifespans from other aging studies, and to a second wild-type included in these experiments – *Oregon R* (*OR-R* in Fig. 1A). *Drosophila* can carry a number of cryptic bacterial or viral infections that affect fitness (Plus et al., 1976). Notably, infection with *Drosophila* Nora virus is common in lab stocks, and this virus has been shown to reduce lifespan (Habayeb et al., 2009). We thus suspected some component of the virome/microbiome of our *iso w^1118^* flies could be affecting our *iso w^1118^* wild-type but not *ΔAMP Chr2* flies. We therefore cleared our flies of their virome/microbiome through bleaching, allowed microbiome recolonization by microbes in the food medium, and assessed the lifespan of the cleaned *iso w^1118^* stock (protocol in the Materials and Methods). At the same time, we screened our *iso w^1118^* flies for a panel of common contaminating viruses; specifically *Drosophila* sigmavirus, *Drosophila* A virus, *Drosophila* C virus and *Drosophila* Nora virus. We did detect *Drosophila* Nora virus (hereafter ‘Nora’) in our *iso w^1118^* flies, but not in our *ΔAMP Chr2* mutants, nor in other stocks included in these experiments. We also detected Nora virus in four other genotypes at different times during our 5 years of study (*AttC^Mi^*, *Bom*^Δ*55C*^, *Group C* and *OR-R*; defined later in the text). In some cases, these stocks were previously Nora-negative, and so were seemingly contaminated from standard fly tipping (Fig. 1B). Strikingly, the bleaching treatment markedly improved the lifespan of all of these stocks to rival their contemporaries, such as *ΔAMP Chr2* flies. The net lifespan reductions for *iso w^1118^* and *OR-R* were ∼39% and ∼23%, respectively, possibly suggesting genetic background effects in susceptibility to Nora virus. We conclude that our wild-type reference had artificially reduced lifespan, likely due to a cryptic Nora virus infection. Although we took care to control for genetic background effects, this stock, at the time, was not an appropriate baseline for comparison. These considerations are in line with recommendations that healthy wild-type stocks should live to median lifespans of 70-90 days (Piper and Partridge, 2018), emphasizing the importance of considering the health of our control stocks prior to making comparisons across stocks/genotypes.

After detecting this contamination in our wild-type flies and seeing the impressive deleterious effect Nora had on lifespan in our hands, we screened various lab stocks for Nora virus. We found that 20 out of 44 arbitrarily selected stocks (both from our lab and others) were Nora positive, though Nora titres were typically found only at low levels under standard rearing conditions. We also checked RNA samples collected from aged Nora-positive flies (aged >3 weeks), finding consistently high Nora titres (Fig. 1C). These stocks also often showed abdominal bloating at older ages, pre-empting mortality (M.A.H., unpublished observation), similar to bloating seen in flies infected with *Drosophila* C virus (Chtarbanova et al., 2014) or after systemic infection with some strains of *Acetobacter* bacteria (Hanson et al., 2022b). We thus suspect Nora virus contamination contributed greatly to the early mortality of our *iso w^1118^* flies in our first lifespan experiments (Fig. 1A), with a more drastic effect than previously reported (Habayeb et al., 2009). However, we will note that we did not intentionally perform Nora infection experiments, and so here we provide only correlation-based evidence.

### Methodology for measuring lifespan and control genotypes

Following these results, we screened all our fly stocks for Nora virus and bleached Nora-positive stocks before use in experiments. Subsequent longevity experiments were carried out with the following conditions: we used our standard food medium (recipe in the Materials and Methods), flipped flies three times per week, with ∼20 flies per vial, using mated males or females (sexes kept separate), and performed lifespan measurements at 25°C unless otherwise specified. As an assay for brain health, we also monitored locomotor competence during aging using the climbing pass rate assay at 5, 40, 50 and 60 days post eclosion (dpe), unless specified otherwise.

To compare lifespan, we first used a Cox mixed model commonly used in the literature. However, we found this statistical method was overly sensitive for our purposes, as even minor differences in lifespan were returned as highly significant (*P*<0.001). In some cases, this was driven by sex×genotype interaction effects. Because we kept males and females in separate vials, we realized that any putative sex×genotype interactions present in our Cox mixed model were indistinguishable from vial effects. This is especially important as a consideration as our AMP mutants are known to suffer dysbiosis with aging (Marra et al., 2021 and see below), compounding the impact of vial-specific microbiome stochasticity (see File S1 for further discussion). Visual inspection of survival curves suggested that, by and large, even significant Cox mixed model variation in survival largely reflected variation around the mean wild-type lifespan, which is expected when performing multiple hypothesis tests (Streiner and Norman, 2011). Thus, we paired our lifespan analyses with one-way ANOVA statistics run on median lifespans per experiment, intended to get a more stringent measure of lifespan differences by placing greater value on effects that were consistent across experiments. As such, we used one-way ANOVA *P*-values by default in the text to report significant lifespan differences.

**Fig. 2.**
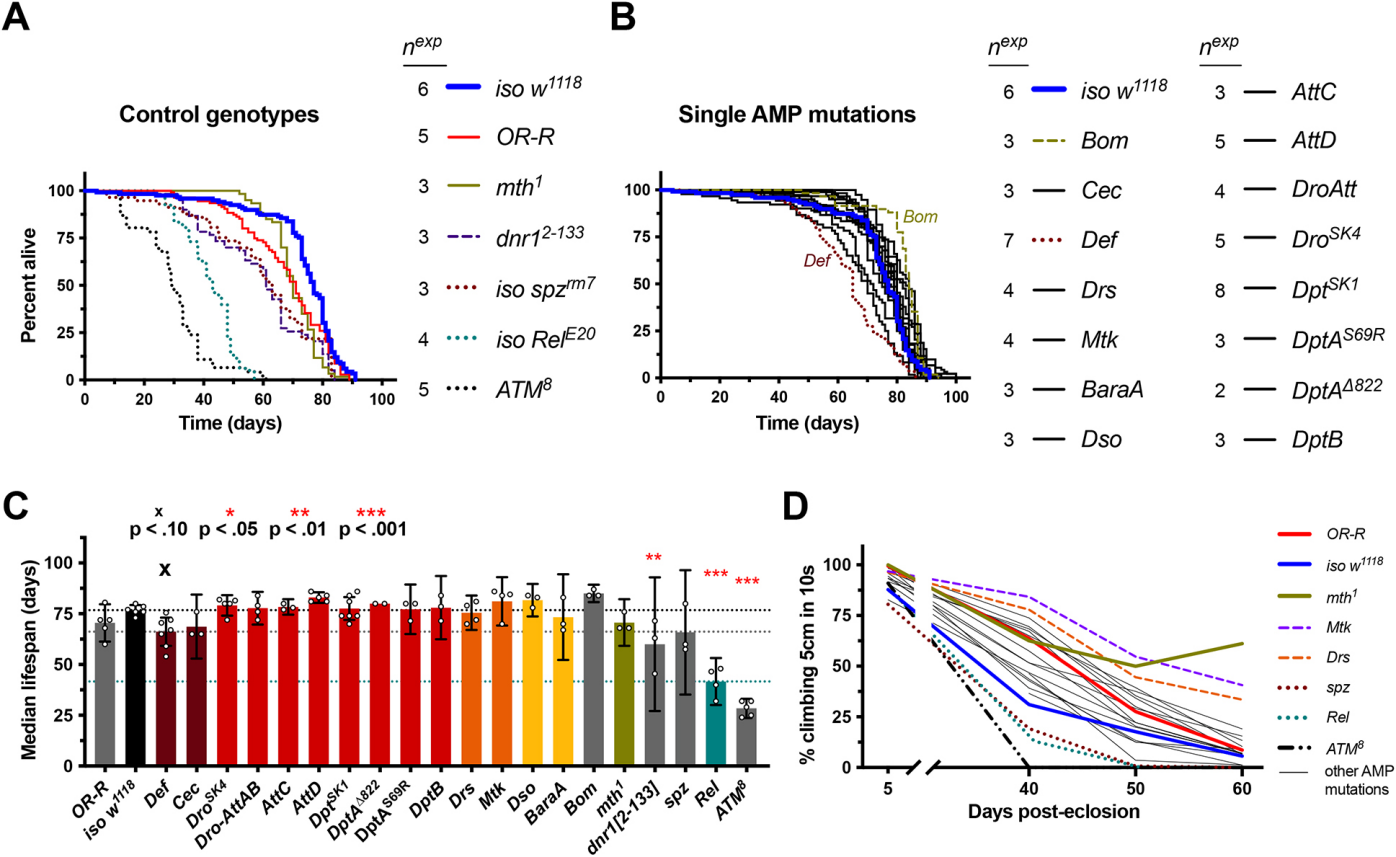
**Individual AMP gene deletions do not drastically affect lifespan.** Results for male flies are reported here and for female flies in Fig. S3. (A) Cumulative lifespans of flies with various genetic backgrounds. Of note, *ATM^8^* data are based on fewer individuals per experiment (see Table S1). (B) Cumulative lifespans of single-gene/single-mutation AMP mutants. Most AMP mutant lifespans (black lines) cluster around the wild-type lifespan (blue line), except *Def^SK3^*. *Bom^Δ55C^* is also noted as an outlier, perhaps living slightly longer than *iso w^1118^*, which was not seen in females (Fig. S3). (C) Median lifespans in which each data point represents one replicate experiment (cumulative of ∼20 males). Median lifespan analysis suggests that the only AMP mutation noticeably differing from *iso w^1118^* is *Def^SK3^*. Of note, the impact of *Def* on lifespan was not corroborated using RNAi (Fig. S4). *Bom^Δ55C^* median lifespans were not different from those of *iso w^1118^*. Horizontal dotted lines indicate median lifespans of *iso w^1118^* (top), *Def^SK3^* (middle), and *Rel^E20^* (bottom). Statistical summaries (^x^*P*<0.1; *P<0.05, **P<0.01, ***P<0.001) reflect comparisons to *iso w^1118^*. (D) Climbing pass rates suggest most AMP mutants climb like wild-type flies, whereas *methuselah* mutants uniquely retain climbing competence into old age (also seen at 29°C, Fig. S2); *Mtk* and *Drs* males also show improved climbing with aging (but see text in File S1).

Before we addressed the impact of AMP mutations on lifespan, we analysed the lifespans of mutants affected in the Toll (*iso spz^rm7^*) or Imd (*iso Rel^E20^*) pathways that have been backcrossed in the DrosDel background (Ferreira et al., 2014). We also compared our DrosDel *iso w^1118^ white*^−/−^ flies with another wild-type (*OR-R*), which has *white^+/+^* red eyes. Of note, *white* gene deletion may affect lifespan through, for example, the role of *white* in the brain (Ferreiro et al., 2017), or through *white-*mediated regulation of intestinal stem cell proliferation (Sasaki et al., 2021). We further included non-isogenic flies with previously reported lifespan effects to show how control genotypes behave in our conditions, and set expectations for the size and consistency of lifespan effects we could reasonably observe in our hands compared to those observed in other studies. These included: *methuselah* mutants suggested to have exceptionally long lifespan (*mth^1^*) (Lin et al., 1998), a *dnr1* mutation that reduces lifespan previously associated with neurodegeneration and aberrant AMP induction (*dnr1^2-133^*) (Cao et al., 2013), and *ATM* mutant flies with precocious neurodegeneration caused by autophagy defects (*ATM^8^*), also associated with Imd pathway activation (Petersen et al., 2013). *mth^1^*, *dnr1^2-133^* and *ATM^8^* flies were not backcrossed into the DrosDel background and had red eyes. Our experiments confirmed that *ATM^8^* flies had a significantly shorter lifespan (Fig. 2A,C) associated with poor climbing competence (Fig. 2D). We also observed that our *OR-R* wild-type displayed a shorter lifespan compared to that of our DrosDel *iso w^1118^* wild-type. Contrary to expectation, the lifespan of *mth^1^* flies was not longer than that of wild-type flies (*P*>0.10, Fig. 2A,C). However, we did observe improved climbing competence of *mth^1^* flies into old age (Fig. 2D), which suggests these flies do have a form of improved fitness with aging in our hands. These experiments also confirmed a reduced lifespan in *Rel^E20^* flies as found by other studies (Kounatidis et al., 2017; Petersen et al., 2013), suggesting a significant contribution of the Imd pathway transcription factor *Relish* to aging.

We also repeated these lifespan experiments at 29°C, which represents a more stressful temperature for *D. melanogaster*, causing more rapid aging (Miquel et al., 1976). When we reared flies at 29°C, as expected, we observed precocious aging in terms of both lifespan and climbing pass rate compared to rates seen in flies reared at 25°C for all genotypes tested: *iso w^1118^*, *OR-R*, *dnr1^2-133^*, *mth^1^* and *Rel^E20^* (Fig. S2A,B). Again, *mth^1^* flies retained climbing competence longer than other genotypes (Fig. S2C), despite no lifespan extension effect. Of note, differences in lifespan between *OR-R* and *iso w^1118^* were lost when flies were raised at 29°C. The previous *dnr1* mutant study showing reduced lifespan used 29°C (Cao et al., 2013), and we found that *dnr1^2-133^* flies generally had reduced lifespan compared to the lifespans of *iso w^1118^* and *OR-R* wild-type at 29°C (Fig. S2B), albeit the lifespans were only significantly different between female *OR-R* and *dnr1^2-133^* flies (*P*<0.05). However, at 25°C, the lifespan of *dnr1^2-133^* flies was not significantly reduced compared to that of *OR-R* flies (*P*>0.10).

Collectively, we observed that *ATM*, *dnr1* and *Relish* deletion reduced lifespan, consistent with previous studies (Cao et al., 2013; Kounatidis et al., 2017; Petersen et al., 2013; Shaposhnikov et al., 2014). Surprisingly, we did not observe lifespan extension in *methuselah* mutants. However, *mth^1^* flies retained climbing competence far better than any other genotype assayed, indicating these flies did have a form of improved aging in our hands. Thus, we could broadly replicate the trends of findings from previous studies, although the lifespan measurements were not always repeatable in our hands.

### Deletion of single AMP genes does not drastically affect lifespan

We next analysed the lifespan of flies lacking individual or small genomic clusters of AMP genes at 25°C. These mutants, for all ‘classical’ AMPs from the seven gene families initially identified, included: a *Defensin* mutant (*Def^SK3^*; referred to as *Def*); deletion of the four *Cecropin* genes *CecA1*, *CecA2*, *CecB* and *CecC* (collectively termed *Cec*); mutation of *Dro* alone (*Dro^SK4^*); mutations of *Dro*, *AttA* and *AttB* (*DroAttAB*); single mutations of *AttC*, *AttD*, *Mtk* and *Drs*; single or combined mutations affecting the two *Diptericins* (*Dpt^SK1^*), *DptA* (*DptA^Δ822^* or *DptA^S69R^*) and *DptB* (*DptB^KO^*); as well as mutants for the more recently described Toll-regulated effector genes *Daisho* (*Dso1* and *Dso2*; collectively *Dso*), *Baramicin A* (*BaraA1* and *BaraA2*; collectively *BaraA*) and *Bomanins* (*Bom^Δ55C^*, indicated as *Bom*; deletions of ten Bom genes at cytogenetic map 55C).

We found no major effect on lifespan of any flies containing single AMP mutations, as survival curves tended to disperse randomly around the *iso w^1118^* lifespan curve, and median lifespans were not significantly different from those of *iso w^1118^* (Fig. 2B,C). The only exception to this trend was male *Def^SK3^* flies, which had noticeably reduced lifespan compared to that of *iso w^1118^* (*P*=0.059). In general, climbing competence was also distributed in a range similar to that of wild-type *iso w^1118^* or *OR-R* flies, and most mutants were not exceptionally good climbers into old age like *mth^1^* flies, though *Mtk* and *Drs* males had somewhat improved climbing into old age (Fig. 2D; Fig. S5). The reduction in lifespan for *Def^SK3^* flies was sufficiently interesting that we tested this effect using ubiquitous (*Actin5C-Gal4*) or glia-specific (*Repo-Gal4*) *Def* interfering RNA (*Def-IR*, RNAi); however, we saw, if anything, the opposite effect when silencing *Def* by RNA interference (RNAi or ‘-IR’) compared to the results seen with the *Def^SK3^* mutant (*Act>Def-IR* males had longer lifespans and females shorter lifespans). However, RNAi genetic controls also suggested complex genetic background effects independent of the Gal4 and RNAi constructs, confounding meaningful interpretations of those results. In general, we could not support a reduced lifespan effect of single AMP mutation with RNAi (Fig. S4).

Collectively, our study using isogenic mutants shows that individual AMP mutations do not significantly affect lifespan, with the possible exception of *Def^SK3^*. Overall, we conclude that deleting single AMP genes has no effect on lifespan beyond levels of difference we also observed when comparing different wild types.

### *ΔAMP14* flies lacking seven AMP gene families display significantly reduced lifespan

**Fig. 3.**
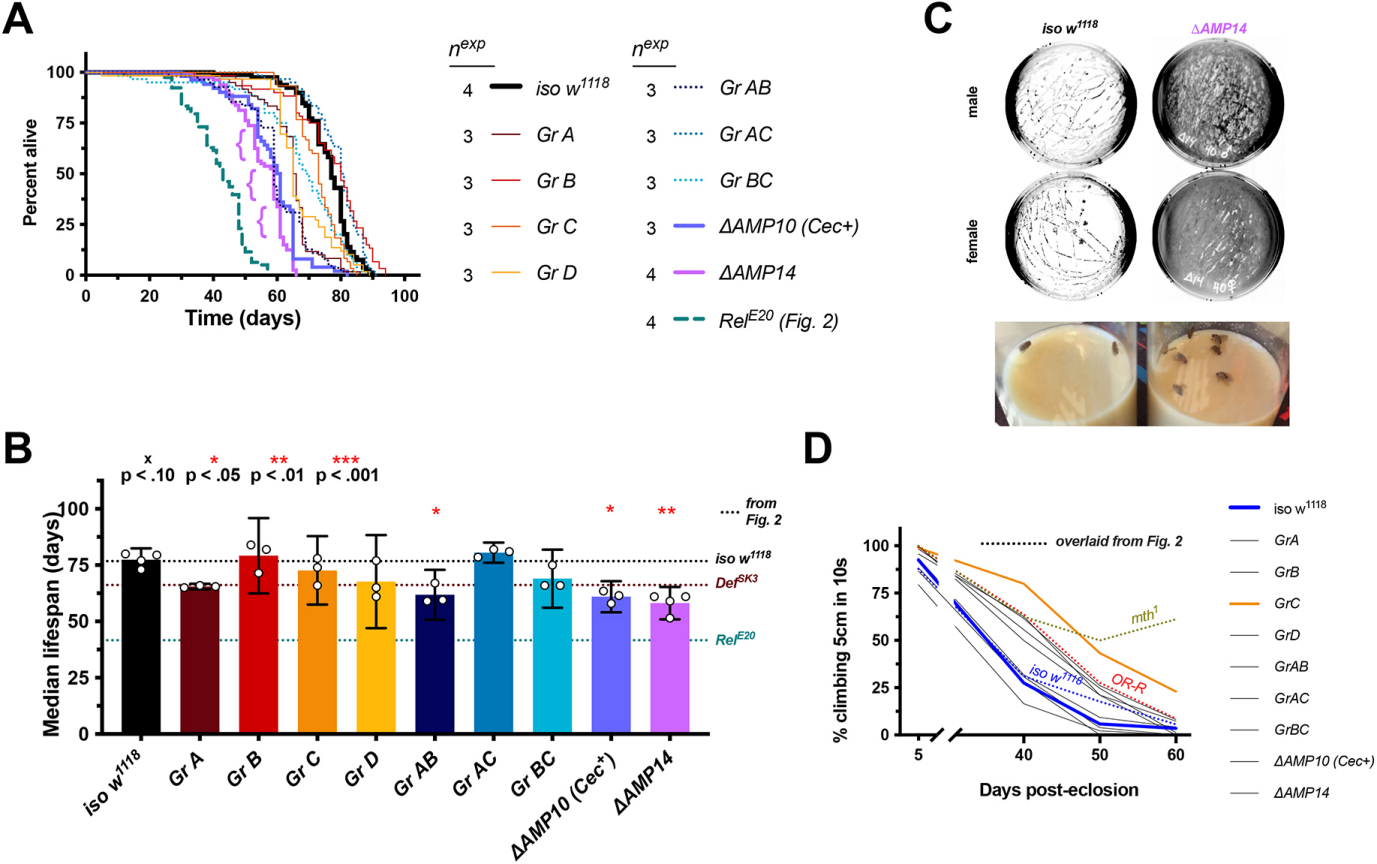
***ΔAMP14* flies have significantly reduced lifespan.** Results for male flies are reported here and for female flies in Fig. S6. (A) Survival curves of various compound AMP mutants. The lifespan of *Rel^E20^* from Fig. 2 is overlaid for direct comparison. ‘{’ annotations highlight major mortality events in *ΔAMP14* flies. (B) Median lifespans of compound AMP mutants. Dotted lines indicate average median lifespans from Fig. 2 of *iso w^1118^* (top), *Def^SK3^* alone (middle), and *Rel^E20^* (bottom) for easier comparisons across figures. Statistical summaries (^x^*P*<0.1; *P<0.05, **P<0.01, ***P<0.001) reflect comparisons to *iso w^1118^* data specific to Fig. 3. (C) Top: representative photonegative images of agar plates seeded by the microbiome found in the vial of 40-day-old flies. Thick bacterial films in *ΔAMP14* vials are readily visualized by this method, which shows the significantly greater bacterial density (dark parts) compared to *iso w^1118^* vials. Bottom: representative photo of *iso w^1118^* and *ΔAMP14* food vials revealing discoloured bacterial biofilm alongside a major mortality event (‘{’ in Fig. 3A). (D) Climbing pass rates of AMP group mutants, with climbing curves from genotypes in Fig. 2 overlaid for direction comparison. *Group C* is highlighted for having a slightly improved climbing over aging, although this improvement is still minor compared to the climbing competence of *mth^1^* flies (also see results for females in Fig. S6 and File S1).

*Drosophila* possess many AMPs with possibly redundant or synergistic activities. Thus, AMPs could affect lifespan only when several genes are deleted simultaneously. After evaluating the effect on lifespan of individual AMP genes or genomic clusters of AMPs, we investigated whether combinatory loss of AMPs could impact aging. Using the same approach as described in Hanson et al. (2019a), we generated four groups of compound AMP mutants that remove different subsets of AMP gene families. *Group A* flies were deleted for *Defensin* and the four *Cecropins*. *Group B* flies were deleted for the structurally related *Drosocin*, *Attacin* and *Diptericin* families. *Group C* flies were deleted for the two antifungal *Drosomycin* and *Metchnikowin* peptide genes. Also, we introduce a new isogenic line combining the loss of two recently described Toll-regulated antifungal peptides: *Group D* flies that were deleted for both *Baramicin A* (Hanson et al., 2021) and the two *Daisho* genes (Cohen et al., 2020). We screened combined mutants lacking each of these AMP groups, including all the combinations of *Groups A*, *B* and *C* [i.e. *AB*, *AC*, *BC* and *ABC* (also known as *ΔAMP14*)]. We also screened flies lacking ten AMP genes, but which retain a wild-type *Cecropin* locus [*ΔAMP10*, as used previously (Carboni et al., 2021; Hanson et al., 2019a)]. In total, we screened nine AMP combinatory genotypes for lifespan and climbing effects (Fig. 3).

We found a significant effect of *Group A* mutations on male lifespan compared to that of *iso w^1118^*, which is not unexpected given this AMP group uses the same second chromosome as the isogenic *Def^SK3^* mutation as above (Fig. 2B), except with the additional loss of Cecropins on the third chromosome. Comparisons between the two suggest a non-significant effect of Cecropin mutation (*Def^SK3^* versus *Group A*, *P*>0.10). Deleting other groups of AMPs typically resulted in non-significant effects on median lifespan compared to their *iso w^1118^* control (*P*>0.10). *Group AB* flies also had reduced lifespan compared to *iso w^1118^*, but were comparable to *Group A* alone (*Group A* versus *Group AB*, *P*>0.10). *Group AC* and *BC* flies had comparable lifespans to those of *iso w^1118^* (*P*>0.10 in both cases), which is notable as *Group AC* flies have the *Def^SK3^* mutation, but only after an additional round of chromosome II recombination with the isogenic *Mtk^R1^* chromosome needed to generate the *Group AC* genotype. Deleting ten AMP genes, which included *Defensin*, also had a *Group A*-like lifespan (*ΔAMP10* versus *Group A*, *P*>0.10). Thus, various combinations of mutants had lifespans comparable to those of either *iso w^1118^* or *Def^SK3^* alone. However, deletion of all 14 classic *Drosophila* AMP genes caused a more pronounced median lifespan reduction than the effect of *Group A* alone (*ΔAMP14* versus *Group A*, male *P*=0.059, female *P*=0.002). There was minor variation in climbing competence amongst AMP mutant groups, with *Group C* flies (*Mtk* and *Drs* double mutants) having slightly improved climbing into old age. However, this effect was minor compared to the climbing competence of *mth^1^* flies into old age (Fig. 3D; File S1).

Although deletion of any single AMP gene had little impact on lifespan, deletion of all 14 classical AMP genes caused a reduction in lifespan that was significantly different from that of wild-type flies. Moreover, recombination of *Def^SK3^* with another mutation to produce *Group AC* flies yielded a lifespan comparable to that of *iso w^1118^.* This result reinforces the need for careful interpretation of the *Def^SK3^* mutation effect of *Group A*. Despite this caveat, *ΔAMP14* flies had significantly reduced lifespan compared to those of both wild-type flies and other AMP groups. The lifespan of *ΔAMP14* flies was intermediate between those of *iso w^1118^* and *Rel^E20^*, indicating *Relish* likely impacts fly lifespan through processes other than AMP regulation.

### *ΔAMP14* flies suffer microbial dysbiosis with aging

During our studies, we noticed that the food surface of the vials containing *ΔAMP14* flies became discoloured and sticky upon aging, suggesting microbial proliferation (Fig. 3C, bottom). The *Drosophila* microbiome is found both in the gut and in the external environment due to constant ingestion and fecal deposition (Broderick et al., 2014; Pais et al., 2018; Storelli et al., 2018). We suspected this change in the fly food appearance for *ΔAMP14* flies upon aging could be linked to a change in the microbiome, as we have recently described a role for AMPs in regulating *Acetobacter* using *ΔAMP14* flies (Marra et al., 2021). Sticky and discoloured food was also observed for *Rel^E20^* flies, which do not express AMPs, and indeed both *ΔAMP14* and *Rel^E20^* flies suffer increased dysbiosis over aging (Marra et al., 2021). To test if microbiome load was associated with early mortality of *ΔAMP14* flies, we monitored bacterial abundance on the food medium. To do this, we emptied vials in which flies had been present for 2 days, added glass beads to these vials, shook the vials with beads for 10 s, then placed the beads on agar plates and spread vial microbes by rolling the beads over the agar for 10 s. By 40 dpe, plating of vial contents using beads confirmed far higher microbe loads in aged *ΔAMP14* vials compared to those in *iso w^1118^* vials (Fig. 3C, top). Moreover, in the time period between 50-70 dpe, most major mortality events in *ΔAMP14* vials were associated with sticky and discoloured bacterial food (example photo in Fig. 3C, bottom). These major mortality events are also seen as precipitous drops in *ΔAMP14* survival curves shown in Fig. 3A (indicated with ‘{’), and similar trends were observed for *ΔAMP10*.

We conclude that *ΔAMP14* flies thus suffered increased microbe load within vials with aging, agreeing with an increase in gut microbiome abundance with aging shown previously using gnotobiotic flies (Marra et al., 2021). This suggests that precocious aging observed in *ΔAMP14* flies could be indirectly caused by the impact of AMPs on the microbiome.

### The lifespan of *ΔAMP14* flies can be rescued significantly by rearing on antibiotic medium

**Fig. 4.**
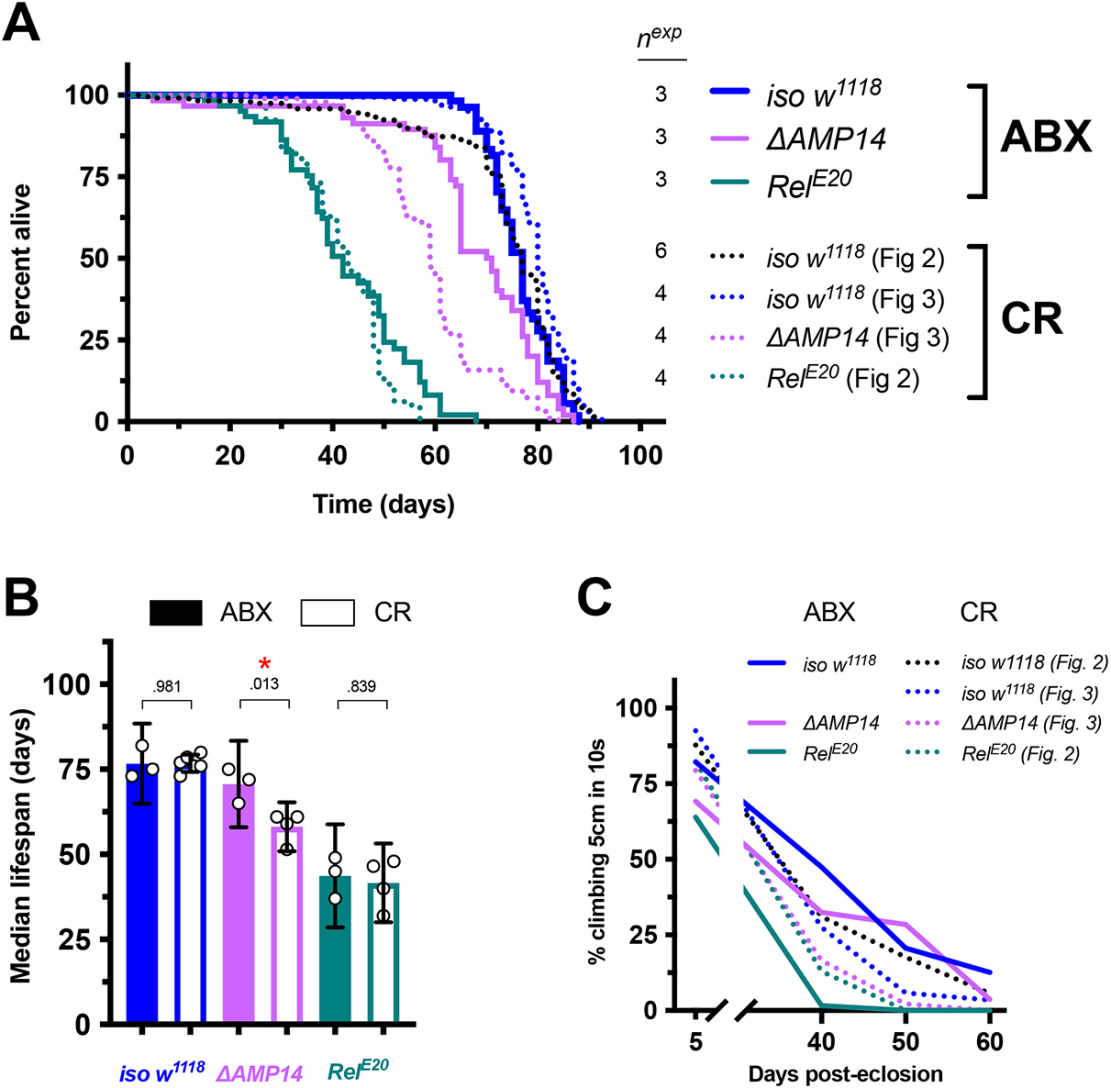
**Microbiome depletion rescues the *ΔAMP14* fly lifespan.** Results for male flies are reported here and for female flies in Fig. S7. (A) Survival curves, including lifespans for both antibiotic-reared (ABX) flies and also conventionally reared (CR) flies from previous figures as dotted lines for direct comparison. (B) Median lifespans, including both ABX and CR fly lifespans for direct comparison (conventionally reared *iso w^1118^* and *Rel^E20^* lifespans shown in Fig. 2C). *P<0.05. (C) Climbing pass rates of ABX (solid lines) and CR (dotted lines) flies at 5, 40, 50 and 60 days post eclosion.

To test whether the effect of AMP mutation on lifespan was linked to changes in the microbiome, we performed the same lifespan experiments in germ-free conditions using *iso w^1118^*, *ΔAMP14* and *Rel^E20^* flies. For this, we first bleached embryos, and then kept larvae and emerging adults on antibiotic (ABX) food media for their entire lifespan. In our hands, antibiotic-reared *iso w^1118^* flies had a similar lifespan compared to conventionally reared (CR) flies. Likewise, in our hands, *Rel^E20^* mutants had similar lifespan regardless of whether they were reared conventionally or in antibiotic conditions. However, rearing *ΔAMP14* flies on antibiotics significantly rescued their lifespan (Fig. 4A,B; *ΔAMP14* ABX versus CR, *P*=0.013), and a similar non-significant trend was seen in females (Fig. S7A,B). Climbing competence remained largely unchanged, although male Δ*AMP14* flies showed improved climbing specifically at 50 dpe in germ-free conditions (*P*=0.053, Fig. 4C).

These results suggest that the short lifespan of *ΔAMP14* flies primarily relies on the impact that AMPs have on the microbiome through aging. The observation that antibiotics treatment rescues *ΔAMP14* but not *Relish* mutant lifespan suggests the short lifespan of *Rel^E20^* flies depends on effects other than the microbiome and independent of AMPs.

## DISCUSSION

A number of studies done not only in *Drosophila* and in *Caenorhabditis elegans*, but also in vertebrates, have implicated AMPs in processes as diverse as behaviour, neurodegeneration, tumour clearance and aging. In mammals, some AMPs function as pro-inflammatory cytokines, and as such could influence these processes by disrupting homeostasis when chronically expressed (Liang and Diana, 2020; Mookherjee et al., 2020; Smith and Gwyer Findlay, 2022). However, to date there is no evidence of a cytokine role for AMPs in *Drosophila* (Hanson and Lemaitre, 2020). AMPs can also disrupt the membranes of aberrant host cells, which may become more negatively charged due to the exposure of phospholipids such as phosphatidylserine, a well-known ‘eat-me’ signal that promotes the phagocytosis of apoptotic cells (Fadok et al., 1992; Hakim-Mishnaevski et al., 2019; Manaka et al., 2004). Studies have now also implicated AMPs in the control of tumorous growth *in vivo* in *Drosophila* (Araki et al., 2019; Parvy et al., 2019), providing a proof of principle that AMP action can target self-tissue. Thus, AMPs could have a role in tissue maintenance with consequences on the aging process. Moreover, AMPs have a number of properties in common with neuropeptides, often being cationic and amphipathic (Augustin et al., 2017; Smith and Gwyer Findlay, 2022), alongside descriptions of AMP-like genes undergoing downregulation in the brain after exposure to pheromones (Gendron et al., 2014) or AMP-like genes modulating sleep (Sinner et al., 2021; Toda et al., 2019), memory (Barajas-azpeleta et al., 2018) and affecting behaviours (Ebrahim et al., 2021; Hanson et al., 2021; Kobler et al., 2020). Thus, it cannot be excluded that AMPs modulate neuronal activity, a process that could impact lifespan.

There was an increase of AMP gene expression observed upon aging, though it was unclear if AMPs directly affect lifespan, or if this activation was just a secondary consequence of aging. For instance, aging is accompanied by dysbiosis and gut barrier dysfunction allowing opportunistic systemic infection by microbiome bacteria (Buchon et al., 2009; Clark et al., 2015; Liu et al., 2022; Marra et al., 2021; Rera et al., 2012), which should activate immune signalling and induce AMP expression. Using flies carrying null mutations in AMP genes, we found no evidence that individual AMPs are so essential to host physiology that they have a notable impact on fly lifespan. Overall, the lifespan of flies mutated for individual genes or clusters of AMPs, Bomanins or Daishos did not differ from that of wild-type. The climbing activities of these mutants were also similar to those of wild-type flies. The only possible exception to this was the apparent lower lifespan of *Def* mutant males. However, conflicting results from RNAi experiments and use of *Group AC* flies with the *Def^SK3^* mutation after additional rounds of recombination suggest the somewhat shortened lifespan of *Def* mutant males was not linked to *Def* mutation itself but due to the presence of one or several cryptic mutations that were not removed during the isogenization process due to their proximity to the *Def* locus. Alternately, complex interactions amongst AMPs could provide a protective effect against *Defensin* mutation, as AMP interactions can synergize to prevent damage to host membranes (Drab and Sugihara, 2020). Overall, our study suggests that individual AMPs do not affect lifespan beyond levels of difference we also saw when comparing wild-type and/or classic aging mutant flies (e.g. *mth^1^*), a result that contradicts other studies using RNAi or overexpression methods. However, our results do support a role for AMPs in regulating the microbiome over aging. Indeed, we recently showed that *Acetobacter* microbiome bacteria grow out of control in the microbiome of Δ*AMP14* flies (Marra et al., 2021), and later confirmed that *Diptericin B* has a highly specific and important role in suppressing *Acetobacter* growth after systemic infection, which causes bloating similar to what we saw in Nora virus-infected flies (Hanson et al., 2022b). This phenotype of *Acetobacter* systemic infection could help explain why flies bloat upon infection by enteric pathogens (like Nora virus or *Drosophila* C virus) or upon aging, given the eventual invasion of gut microbes into the hemolymph (Clark et al., 2015; Rera et al., 2012).

We cannot exclude that additional AMP mutant combinations could reveal a stronger impact of AMPs on lifespan. Indeed, a number of immune-induced peptides that could be AMPs await description (Hanson and Lemaitre, 2020; Schlamp et al., 2021), and additional deletion of these peptides could explain the difference in lifespan between *ΔAMP14* and *Relish* mutants. Use of other rearing conditions could also reveal a role for AMPs not found here. As part of this study, we also monitored AMP expression upon aging in four wild-type backgrounds (a lab standard *w^1118^*, *Exelexis*, *OR-R* and *Canton-S*) by separating out dissected heads and bodies (thorax and abdomen). In our hands, we observed increased expression of AMP genes in fly bodies aged 40 days compared to expression seen in young flies, though the extent differed by genotype (Fig. S9; Table S2). However, we saw no marked AMP increase in the head with aging, including in a separate experiment following flies over multiple time points (Table S2). Indeed, when AMPs were upregulated in the head upon aging, this was often coupled with a stronger upregulation in the body, suggesting that this increase of AMP expression in the head could derive from the head-specific fat body responding like the fat body in the thorax and abdomen. We also tested if glia-specific *Relish* knockdown (via *Repo-Gal4* and *UAS-Rel-IR*) could rescue lifespan, as many studies suggest a role of *Relish* in neurons/glia to rescue neurodegenerative syndromes (Cao et al., 2013; Kounatidis et al., 2017; Petersen et al., 2013; Shukla et al., 2019), but, if anything, we saw the opposite effect: female *Repo>Rel-IR* flies had shorter lifespan than genetic controls, and we found that *Repo-Gal4* alone had improved climbing into old age, suggesting a genetic background effect on aging unrelated to RNAi using this driver (Fig. S8). Although *Repo>Rel-IR* can rescue disease phenotypes in models of neurodegeneration, taken together, our results suggest AMPs are not especially upregulated in the head upon aging (except when also upregulated in the body) and that glia-expressed *Relish* does not have a major deleterious role in lifespan using our standard rearing conditions. Differences in flies between research groups (local microbiome composition, including cryptic viral infections, food recipe, etc.) could account for these conflicting results. As we found by including *methuselah* mutant flies, improvements to healthy aging could also be consistent across research groups, but actual lifespan extension may be lab specific. These factors, and the importance of assaying multiple healthy aging metrics, should be considered when comparing our AMP mutant results to those from the larger field.

Importantly, our study suggests that AMPs collectively affect fly lifespan through their impact on the microbiome. Several studies have investigated the role of the gut microbiome on lifespan using germ-free conditions with mixed results (Brummel et al., 2004; Cai et al., 2021; Iatsenko et al., 2018; Ren et al., 2007; Shukla et al., 2021). It could be that discrepancies between studies reporting an impact of the microbiome on aging results from a complex interaction between nutrition and gut bacteria. In poor or unbalanced diets, the microbiome could have a positive impact on lifespan by extracting more nutrients from the food (Camus et al., 2019; Chaston et al., 2016; Consuegra et al., 2020; Erkosar et al., 2017; Newell and Douglas, 2014; Storelli et al., 2018; Téfit et al., 2018). In contrast, on a lab-standard nutrient-rich diet, as we have used in this study, the microbiome could have less impact. Consistent with this, we did not see major differences between germ-free and conventionally raised wild-type flies in this study. Previous studies have already revealed the role of Imd signalling in controlling microbiome load and diversity, preventing dysbiosis (Guo et al., 2014; Li et al., 2016; Liu et al., 2022; Yamashita et al., 2021). The specific microbiome of flies from a given research group could also change the impact of germ-free conditions. For instance, different *Acetobacter* strains have different virulence to the fly during systemic infection (Hanson et al., 2022b), which accompanies aging and intestinal barrier dysfunction (Clark et al., 2015; Rera et al., 2012). Indeed, use of *ΔAMP14* flies revealed a key role of AMPs to control *Acetobacter* levels in the microbiome and, accordingly, *ΔAMP14* flies have increased *Acetobacter* loads upon aging (Marra et al., 2021).

Our present study shows that the action of AMPs preserves lifespan, and that this effect is largely due to their impact on the microbiome. Thus, the impact of AMPs on lifespan is consistent with their well-established microbicidal activity. Interestingly, raising *Relish* mutant flies in axenic conditions did not lead to lifespan extension, indicating that *Relish*, and likely the Imd pathway, have a much more profound impact on host physiology independent of AMP regulation. In line with this, *Relish* and the Imd pathway have been implicated in neural systems (Kounatidis et al., 2017; Masuzzo et al., 2019; Shukla et al., 2019), cell competition (Meyer et al., 2014; Nandy et al., 2018), metabolism (Molaei et al., 2019; Musselman et al., 2018) and enterocyte delamination (Liu et al., 2022; Zhai et al., 2018), processes that likely impact lifespan. It is interesting to note that the food media of *ΔAMP14* and *Rel^E20^* flies were enriched in bacteria, agreeing with elevated bacterial loads in these flies with aging as shown previously (Marra et al., 2021). External microbes colonize the *Drosophila* gut, and gut microbes are released into the external environment as part of excreta (Broderick et al., 2014; Newell and Douglas, 2014; Storelli et al., 2018; Winans et al., 2017). Thus, it was expected that an increased gut microbiome load in the absence of AMPs would result in high bacterial load in the fly food medium. Although we reared flies in a vial in artificial lab conditions here, it is tempting to speculate that AMPs expressed in the gut could not only shape the gut microbiome but also environmental bacteria. The increased load in the food medium could therefore rely either on AMP-mediated control of the gut microbiome or on external AMPs secreted into the food medium. This would suggest that AMP mutation can exacerbate microbiome effects from both within the fly and in the external vial environment.

During our study, we experienced a number of challenges to lifespan data interpretation. Notably, our reference wild-type and several other fly strains were infected with *Drosophila* Nora virus, which had a greater deleterious effect on lifespan of *iso w^1118^* compared to wild-type flies used in a previous study (Habayeb et al., 2009). Thus, we initially interpreted our early results as if AMP deletion extended lifespan to a great extent compared to that of the isogenic wild-type controls (Fig. 1A). However, our ultimate findings, including standard genotypes and different conditions, instead highlight that loss of AMPs does not extend lifespan; if anything, they show the opposite. We also note that cryptic and chronic infections common in fly stocks, such as Nora virus, represent a serious threat to aging studies. In our study, we realized this cryptic viral infection confounded our results when comparing the lifespan of our isogenic wild-type flies to the expected absolute lifespan of *Drosophila* according to previous recommendations (Piper and Partridge, 2018). We publish this experience, which confused years of data collection, as a cautionary note for the field of aging and immunity. Our hope is that our experience can help others avoid similar confounding factors.

In conclusion, our study reveals a key role of AMPs in the aging process, but mainly through their indirect effect on the microbiome. We cannot exclude that certain contexts could reveal an intrinsic effect of AMPs on host tissues during aging, such as conditions found in individuals that have aging-associated diseases like cancer or precocious neurodegeneration, uncommon in standard wild-types. However, here we did not find evidence of AMPs directly impacting aging in a striking way. We are still far from understanding the complex relationship between the immune system, senescence, and aging, which requires further investigation.

## MATERIALS AND METHODS

### *Drosophila* rearing conditions

*Drosophila* stocks used in this study, including genotype descriptions, are listed in Table S3. Food media used the following recipe (per 600 ml): 3.72 g agar, 35.28 g cornmeal, 35.28 g yeast extract, 36 ml grape juice, 2.9 ml propionic acid and 15.9 ml Moldex. The antibiotic medium also contained final concentrations of 50 μg/ml ampicillin, 50 μg/ml kanamycin, 10 μg/ml tetracycline and 10 μg/ml erythromycin. Flies were flipped three times per week (Monday, Wednesday, Friday), and vials were left on their side to ameliorate the effect of the food medium stickiness on mortality with aging by allowing flies falling to the ground to drop onto plastic rather than the food surface. This precaution was taken as vial conditions differ markedly in specific immune-deficient genotypes (Marra et al., 2021).

To clear flies of Nora virus, embryos were collected from grape juice agar plates, rinsed with distilled water and left to soak in 3% bleach for 3 min. Embryos were then rinsed twice in distilled water for 1 min each. This protocol was also used to clear the microbiome of antibiotic-reared flies, whereafter we placed embryos directly on antibiotic medium for germ-free experiments.

### Lifespan experiments

Lifespan experiments were conducted from 2017 to 2022. Flies were allowed to emerge and mate randomly for ∼3 days prior to separating males and females. Then, groups of 20 males or 20 females (mated) were flipped three times per week (Monday, Wednesday, Friday) to measure fly lifespan in 90×15 mm polystyrene vials.

We used a Cox proportional hazard (CoxPH) mixed-effects model to initially analyse lifespan effects, with experimental replicate and biological sex as interaction terms in R version 3.6.3. In both cases, experimental replicate and sex were significant contributors to the model (*P*<0.001). Our impression from the initial data analysis was that the CoxPH model was overly sensitive to minor variation around the geometric mean lifespan of *iso w^1118^* control flies, exacerbated by the large sample sizes used and the many comparisons performed in our study inflating the likelihood of type I (false-positive) statistical errors. Even if minor differences in lifespan were genuine, their ultimate importance was questionable when compared to other genetic backgrounds (e.g. *OR-R*, *mth^1^*), particularly given variable mutation types and transgene insertions used for different AMP mutations (i.e. point mutation, genomic deficiency, *white^+^*, 3×P3-EGFP or 3×P3-dsRed). We thus preferred to focus on each experiment as if that population of flies represented a single sampling. We therefore treated our data as if we had, for example, *n*=3 per genotype (three experiments), rather than *n*=120 (60 males and 60 females) per genotype across three experiments. For this purpose, we decided to use median lifespans as our primary readout. Sex-specific median lifespans were analysed using one-way ANOVA with Holm–Sidak's multiple test correction implemented in Prism v9.3.1, or one-way ANOVA with Tukey's honestly significant difference (HSD) correction in R v3.6.3.

### Climbing pass rates

We paired our lifespan data with the gravitaxic locomotor climbing assay to provide an independent metric of aging (Madabattula et al., 2015). This assay assesses the general locomotor competence of the fly, which is often used as a readout of neurodegeneration, but can also reflect generic aging effects (e.g. muscle weakness). Climbing pass rates were filmed for flies at 5, 40, 50 and 60 dpe ±2 days, and analysed later manually. A pass was considered if a fly climbed 5 cm within 10 s of being tapped to the bottom of the vial. Flies were transferred to a chamber made of two empty vials stacked atop each other to provide ample room to climb upwards without reaching the ceiling or disrupting other flies. Two sets of broken markings were made with permanent marker at 2.5 cm and 5 cm on the lower vial as reference points, though we ultimately report only 5 cm climbing rates given similar trends between the two. At each time point, an initial tapping down was performed to associate the flies with their new environment and encourage climbing behaviour, as we found the first repetition of this experiment often had fewer climbers than subsequent repetitions. After this initial association, three technical repeats of the climbing assay were performed, and final values represent the average of these three technical repeats. All climbing measurements were taken between 2 pm and 5 pm to ensure a consistent measurement timeframe.

This assay can be a measure of both climbing speed and/or readiness of response: for instance, older flies sometimes suffered temporary seizures after being tapped down, or might climb slower, more erratically, or simply to a lesser extent than younger counterparts. In most cases, climbing pass data mirrored lifespan data in terms of relative trends. We thus have no suspicion that AMP mutations, individually or collectively, greatly affected locomotory competence with aging. The only noteworthy exception to mirrored trends between lifespan and climbing was *methuselah* mutant flies, which had wild-type-like lifespans, but improved climbing competence into older age at both 25°C and 29°C.

For analysis of trends of *ΔAMP14* in Fig. 4C, we fit a two-way ANOVA measuring the climbing at 40, 50 and 60 days, with an interaction term for age and germ-free conditions in Prism v9.4.1.

### Microbiome monitoring

We monitored the microbiome of compound AMP mutants and antibiotic-reared flies by checking plated vial food contents on MRS+mannitol agar, a medium amenable to both Lactobacilli and *Acetobacter*. Specifically, at both ∼20 and ∼40 days, we took vials in which flies had been present for 2 days, added five glass beads to the vials, shook the glass beads in vials for 10 s, then transferred glass beads to MRS+mannitol agar plates and shook the plates for 10 s, then removed the beads and left plated microbes to grow overnight at 29°C. Vial microbiome loads were checked on Wednesdays. We chose Wednesdays rather than a precise age (e.g. exactly 20 dpe) to ensure that flies entered the plating time point after experiencing a similar regimen of flipping; i.e. in our experiments, we only plated microbes from vials in which flies had spent the past five days with ∼3 days in a vial accruing microbes over the weekend (Friday to Monday), and the next two days depositing microbes in the vial that was ultimately measured (Monday to Wednesday). This design was chosen based on demonstrations that flipping regimen drastically affects microbiome load (Arias-Rojas and Iatsenko, 2022; Blum et al., 2013; Pais et al., 2018). Beyond ∼40 days, comparisons were not equal due to onset of mortality in AMP mutants and associated drops in vial fly density.

In experiments using this method with antibiotic medium, microbes were never detected from overnight growth to monitor vial microbiome loads.

### Gene expression assays

Gene expression was performed using primers listed in Table S3 with PowerUP SYBR Green Master Mix (Applied Biosystems), using the Pfaffl method of quantitative PCR (qPCR) quantification with Rp49 as the reference gene (Pfaffl, 2001). RNA was extracted using TRIzol (Invitrogen) according to manufacturer's protocol. cDNA was reverse transcribed using Takara Reverse Transcriptase.

Dissections of heads from bodies were performed in ice-cold PBS, and tubes containing pools of 20 heads or bodies were kept at −20°C until after TRIzol was added to prevent RNA degradation before sample processing.

## Supplementary Material

10.1242/dmm.049965_sup1 Supplementary information
