# Supplementary material for: Antimicrobial peptides do not directly contribute to aging in Drosophila, but improve lifespan by preventing dysbiosis
Source: Dis Model Mech. 2023 Apr 26;16(4):dmm049965. doi: 10.1242/dmm.049965 (PMC10163324; doi:10.1242/dmm.049965)
Supplement: Supplementary information [file dmm-16-049965-s1.pdf]

Fig. S1

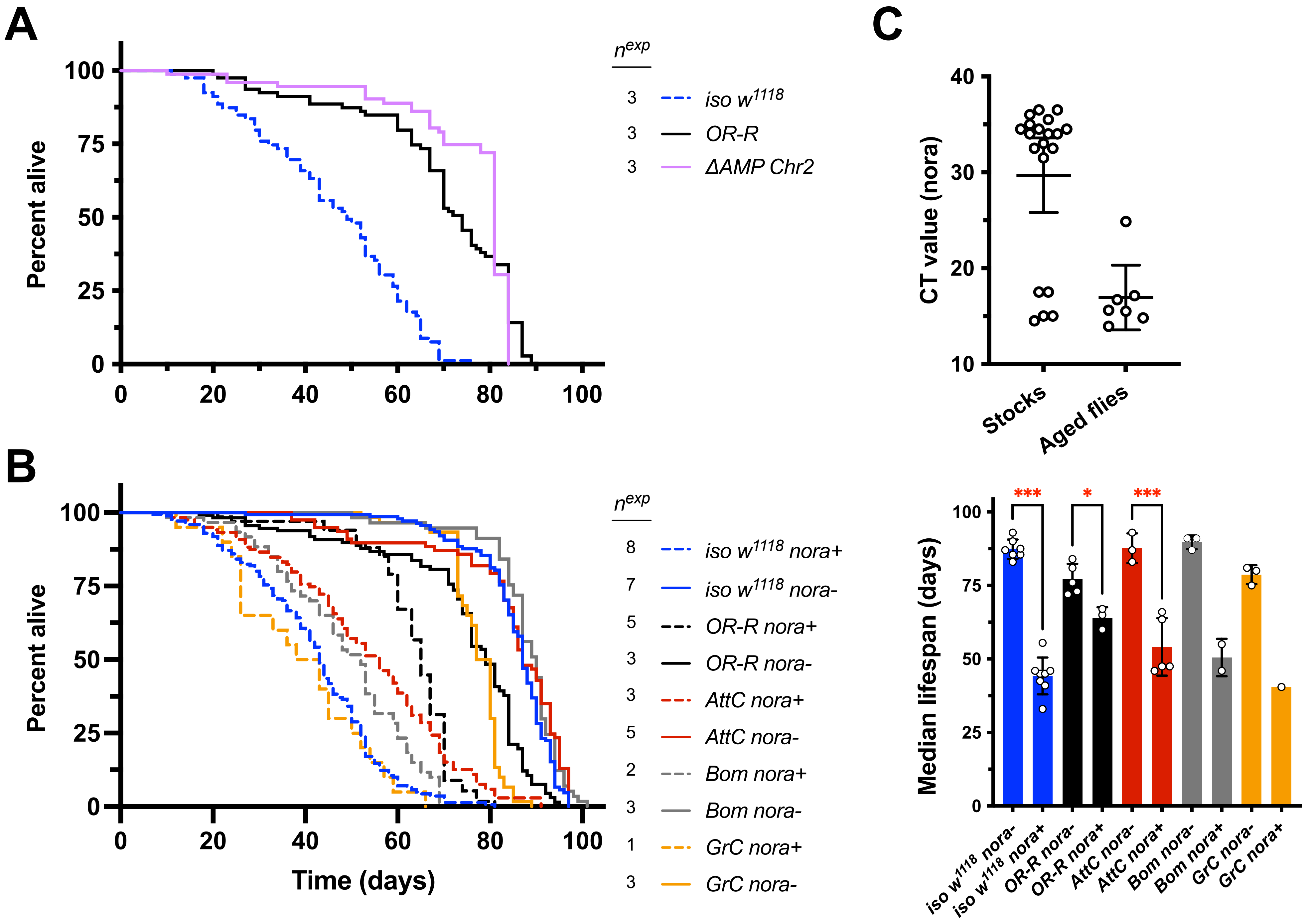


**Fig. S1. Lifespan of various fly genotypes at 29°C. Male flies are shown in Figure 1.** A) Comparison of lifespan from early experiments using two wild-types (*iso* $w^{1118}$ and *OR-R*) alongside compound AMP mutants lacking *Def, Dro, AttA,B,C, Mtk,* and *DptA,B*, which are deleted in *ΔAMP Chr2* flies. B) Effect of *nora* clearance on lifespan of *iso* $w^{1118}$*, OR-R, AttC, Bom,* and *GrC* genotypes. Median lifespans are shown in the right panel (** P < .01, *** P < .001). Number of independent experiments ($n^{exp}$) is reported. C) *Nora* titres measured by CT value in 18 °C source stocks (Stocks) or *nora*-positive flies aged 3+ weeks kept at densities of 20 flies per vial (Aged flies). CT values represent nora titre from 5ng total fly RNA per 10µL qPCR reaction.

Fig. S2

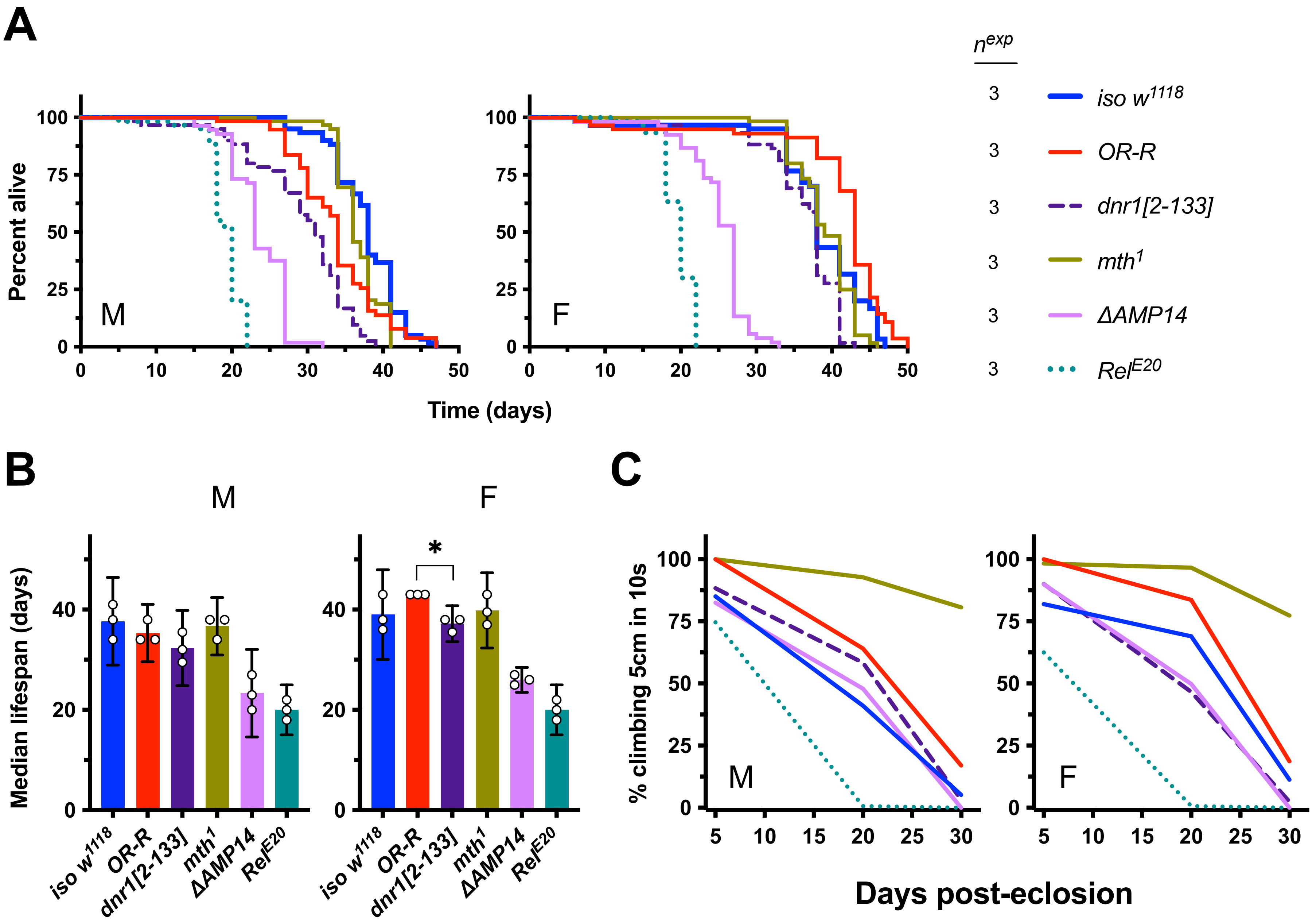


**Fig. S2. Lifespan of various fly genotypes at 29°C.** A) *Relish* mutant (*$Rel^{E20}$*) and compound AMP mutants (*ΔAMP14*) suffer significantly reduced lifespan at 29°C. B) Median lifespan data from Figure S2A. C) Climbing pass rates of flies reared at 29°C. *$mth^1$* flies are the only fly line that retains climbing competence into old age.

Disease Models & Mechanisms • Supplementary information

Fig. S3

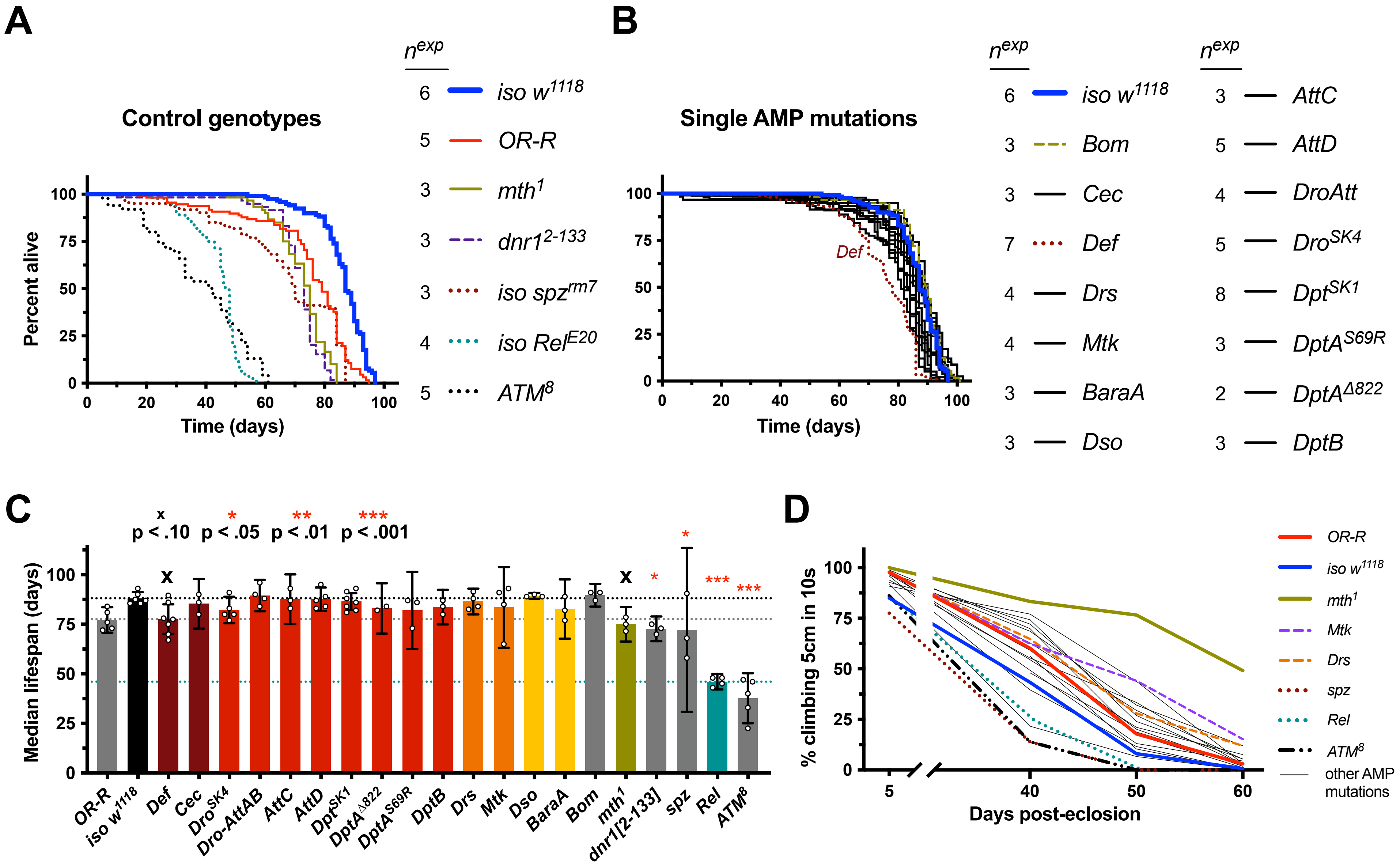


**Fig. S3. Individual AMP gene deletions do not drastically affect lifespan.** Male flies shown in Figure 2. A) Cumulative lifespans of female flies with various genetic backgrounds. Of note, $ATM^8$ data are based on fewer individuals per experiment (see File S1). B) Cumulative lifespans of single gene AMP mutants. Most AMP mutant lifespans (black lines) cluster around the wild-type (blue line), except $Def^{SK3}$. C) Median lifespans where each data point represents one replicate experiment (cumulative of 20 females). Median lifespan analysis suggests that the only AMP mutation statistically differing from *iso* $w^{1118}$ was $Def^{SK3}$. Of note, the impact of *Def* on lifespan was not corroborated using *Defensin* RNAi (Fig. S4). Horizontal dotted lines indicate median lifespans of *iso* $w^{1118}$ (top), $Def^{SK3}$ (middle), or $Rel^{E20}$ (bottom). Statistic summaries (p-values: ˣ, *, **, ***) reflect comparisons to *iso* $w^{1118}$. D) Climbing pass rates suggest most AMP mutants climb like wild-type flies, while *methuselah* mutants uniquely retain climbing competence into old age (also seen at 29°C, Fig. S2).

Disease Models & Mechanisms • Supplementary information

Fig. S4

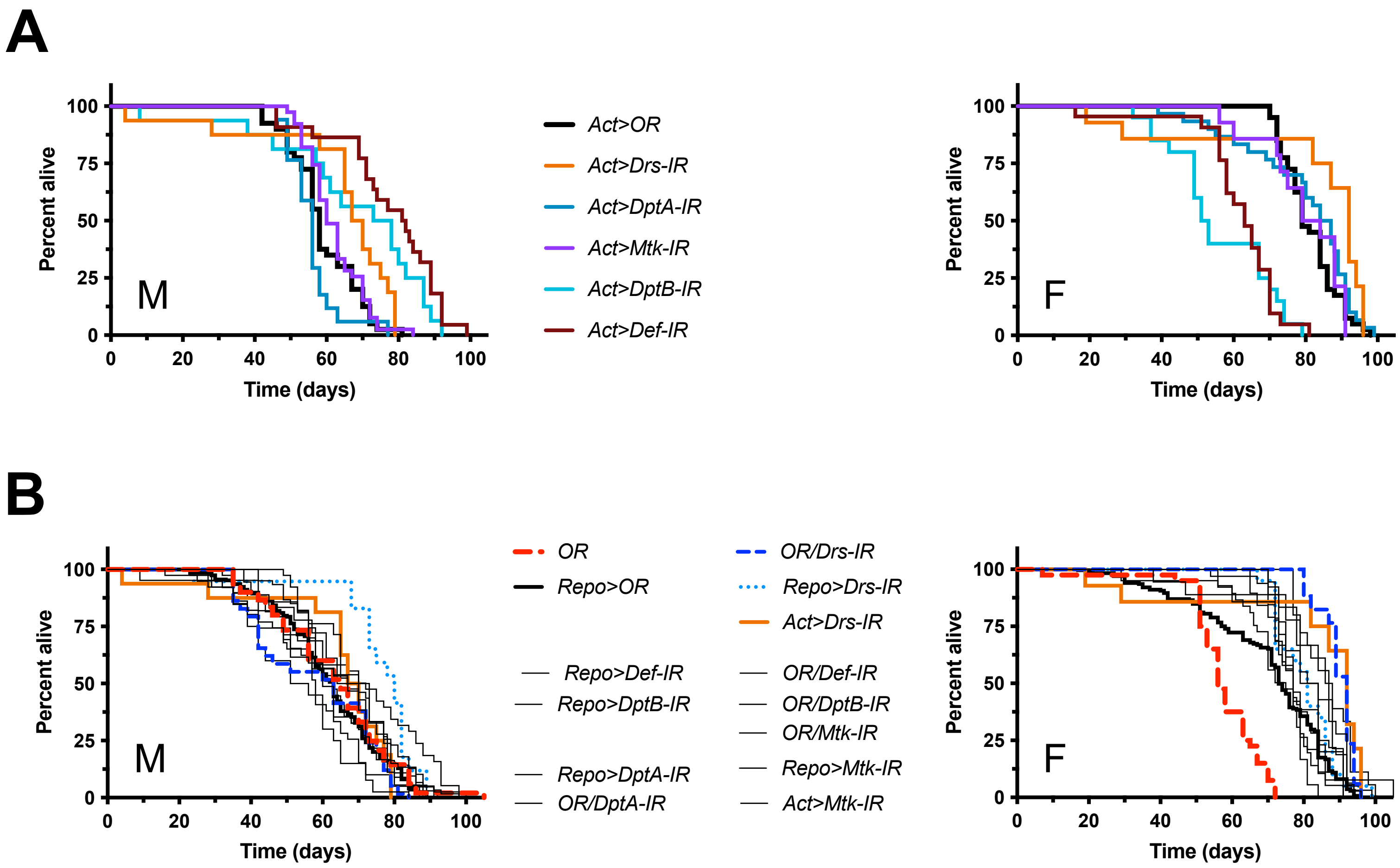


**Fig. S4. AMP gene silencing ubiquitously or in glia emphasizes the importance of reference genotypes for lifespan context.** Males (M) and females (F) shown in separate panels. A) Comparison of multiple *Actin5C-Gal4>AMP-IR* lines shows possible major lifespan effects of Act>Def-IR, with males having extended lifespan compared to controls, while females had reduced lifespan. However these data are exactly opposite to trends from *Def*$^{SK3}$ mutant data, where males had reduced lifespan (Fig. 2) and females had comparable lifespan to wild-type (Fig. S3). B) In general, lifespan differences are not especially striking in the context of additional genetic background controls and references. For instance, female *Act>Drs-IR* lifespan is not very different from *OR/Drs- IR* control, suggesting the *Drs-IR* genetic background is long-lived independent of *Drs* knockdown. Similarly, despite a seeming lifespan extension effect by *Act>DptB-IR* in males, we found no lifespan effect in *Dpt*$^{SK1}$ or *DptB* mutants, both deficient for *DptB* (Fig. 2).

Disease Models & Mechanisms • Supplementary information

Fig. S5

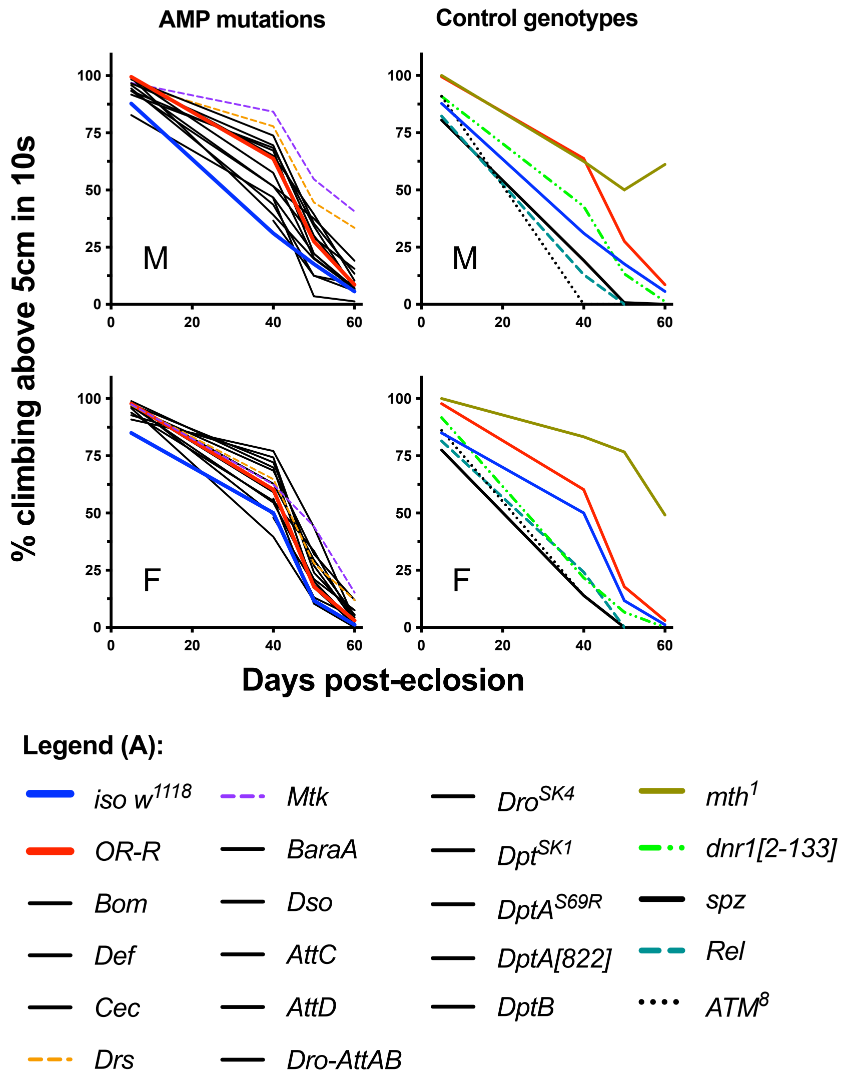


**Fig. S5. Climbing pass rate data overview from individual mutants.** Males (M) and females (F) shown in separate panels. A) Climbing pass rate curves over aging in males and females. AMP mutants displaying climbing rate within wild-type range are left with solid black lines (left panels). Of note, *Drs*$^{R1}$ and *Mtk*$^{R1}$ are the only mutations which are caused by the insertion of *white*$^{+}$ transgenes. Thus, we cannot exclude that their improved climbing effect results from the presence of *white*$^{+}$ compared to *iso w*$^{1118}$ and other AMP mutants. In line with this idea, *OR-R white*$^{+/+}$ wild-type flies similarly trended towards better climbing competence than *iso w*$^{1118}$ flies, although many factors likely distinguish these two genetic backgrounds.

Disease Models & Mechanisms • Supplementary information

Fig. S6

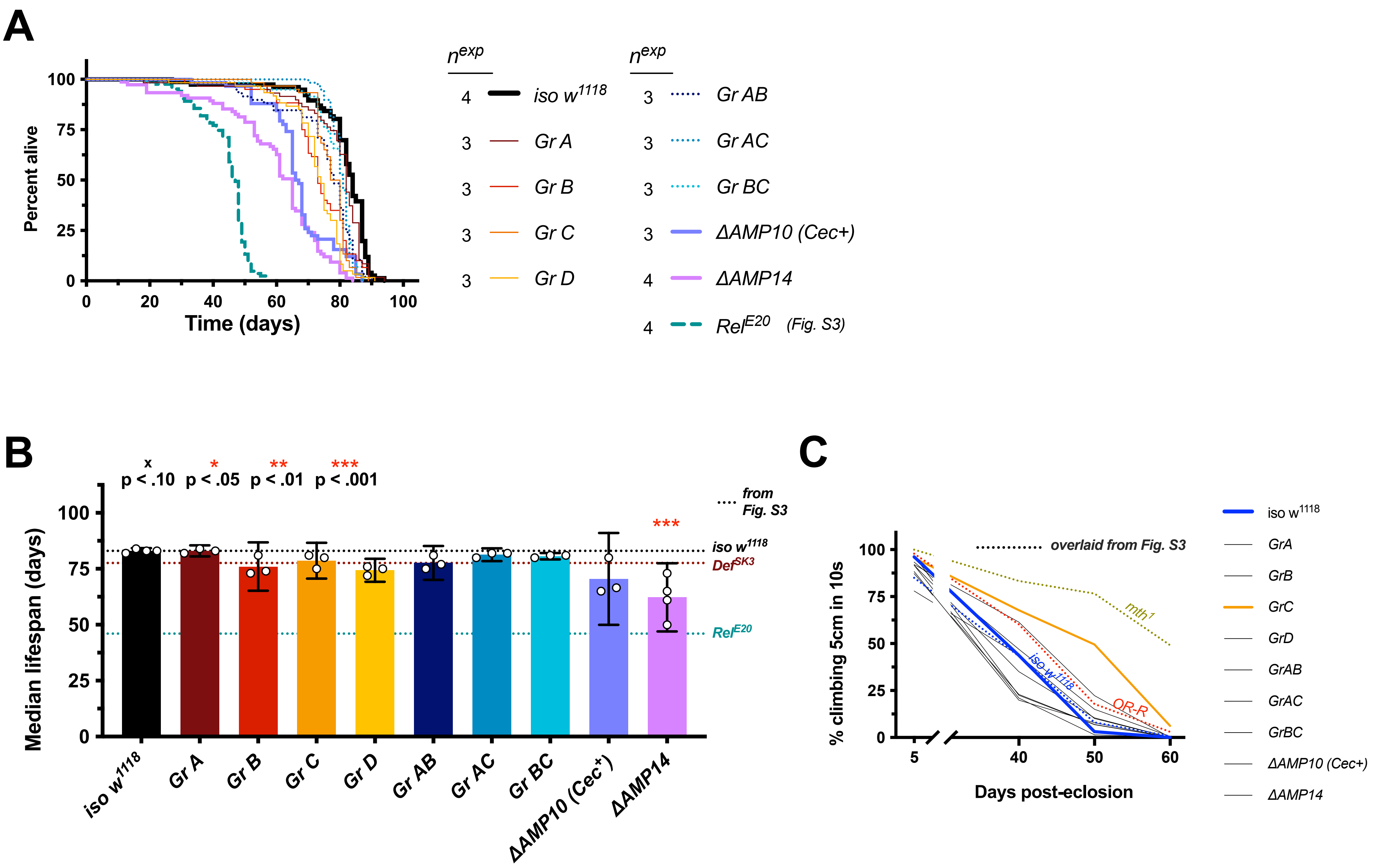


**Fig. S6. *ΔAMP14* flies have significantly reduced lifespan.** Male flies are shown in Figure 3. A) survival curves of various compound AMP mutants. The lifespan of *Rel*$^{E20}$ from Fig. S3 is overlaid for direct comparison. B) Median lifespans of compound AMP mutants. Dotted lines indicate average median lifespans from Fig. S3 of *iso w*$^{1118}$ (top), *Def*$^{SK3}$ alone (middle), and *Rel*$^{E20}$ (bottom) for easier comparisons across figures. Statistic summaries (p-value: ˣ, *, **, ***) reflect comparisons to *iso w*$^{1118}$ data specific to Figure S6. C) Climbing pass rates of AMP group mutants, with climbing curves from genotypes in Fig. S3 overlaid for direction comparison. *Group C* is highlighted for having a slightly improved climbing over aging, though this improvement is still minor compared to the climbing competence of *mth*$^{1}$ flies (but see Fig. S5 caption and File S1).

Fig. S7

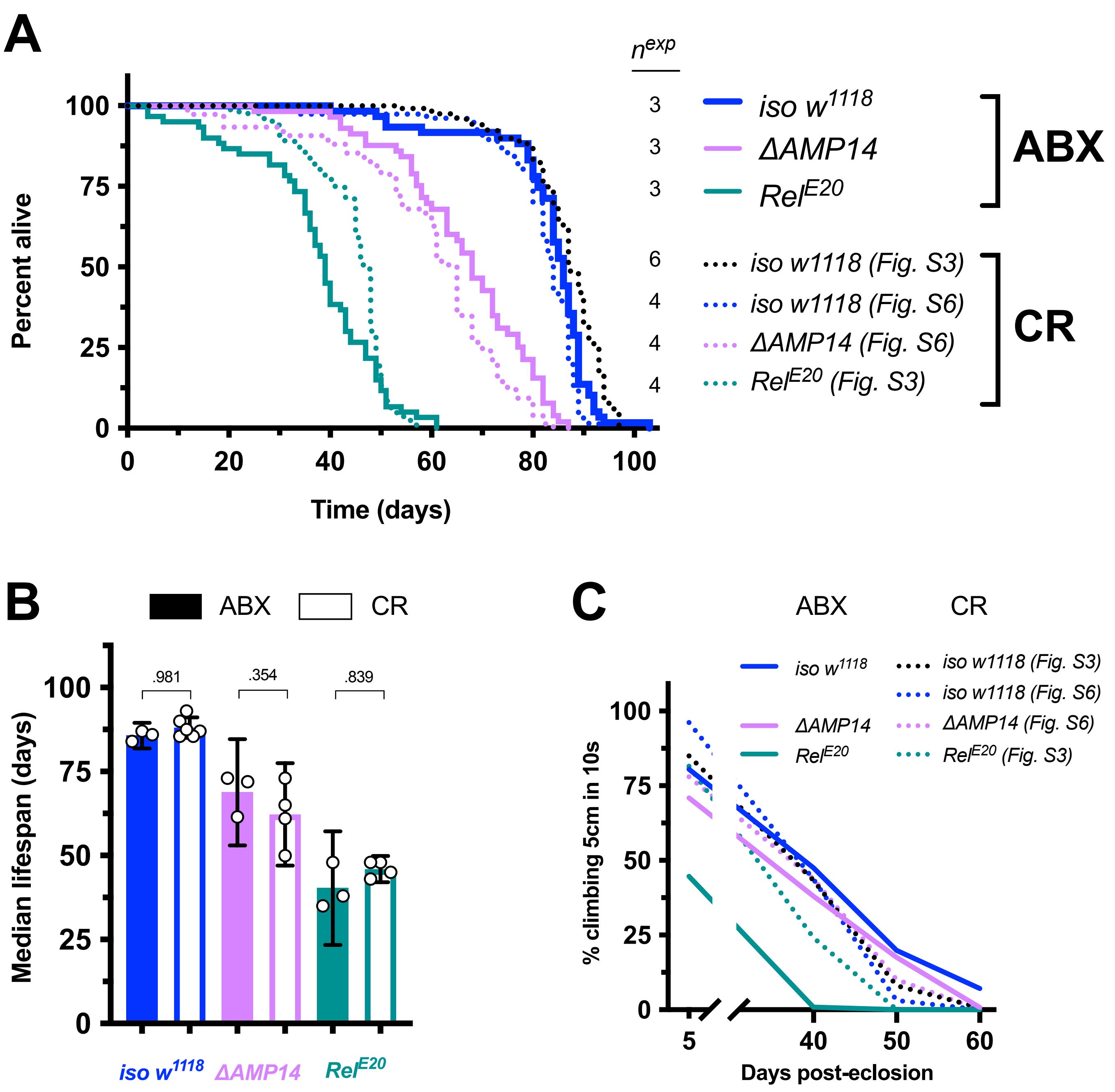


**Fig. S7. Microbiome depletion rescues the *ΔAMP14* fly lifespan.** Female flies are reported here and male flies in Figure 4. A) Survival curves, including both antibiotic- reared flies (ABX), and also conventionally-reared (CR) lifespans from previous figures as dotted lines for direct comparison. B) Median lifespans, including both ABX and CR fly lifespans for direct comparison. (conventionally-reared *iso* $w^{1118}$ and $Rel^{E20}$ lifespans shown in Fig. 2C). C) Climbing pass rates of ABX (solid lines) and CR (dotted lines) flies at 5, 40, 50, and 60 days post-eclosion. Of note, antibiotic treatment did not rescue female Δ*AMP14* lifespan to the same extent as in males *(*Δ*AMP14-ABX vs.* Δ*AMP14-CR*, P = .354). The general trend of the antibiotic treatment remains the same as in males: Δ*AMP14* flies lived longer in ABX conditions than CR flies (consistent with visual inspection in Figure S7A). Indeed, using a more standard CoxPH mixed model, the rescue effect of antibiotics in Δ*AMP14* females is significant ($P$ = .004). Dissecting the effect at the level of sex*genotype interactions in our study should still be interpreted with caution, as any sex*genotype interaction is indistinguishable from 'vial effects' due to our experimental design, particularly important as microbiome development in conventionally-reared AMP mutants in more stochastic than control flies (Marra et al., 2021). A full discussion is provided in File S1.

Fig. S8

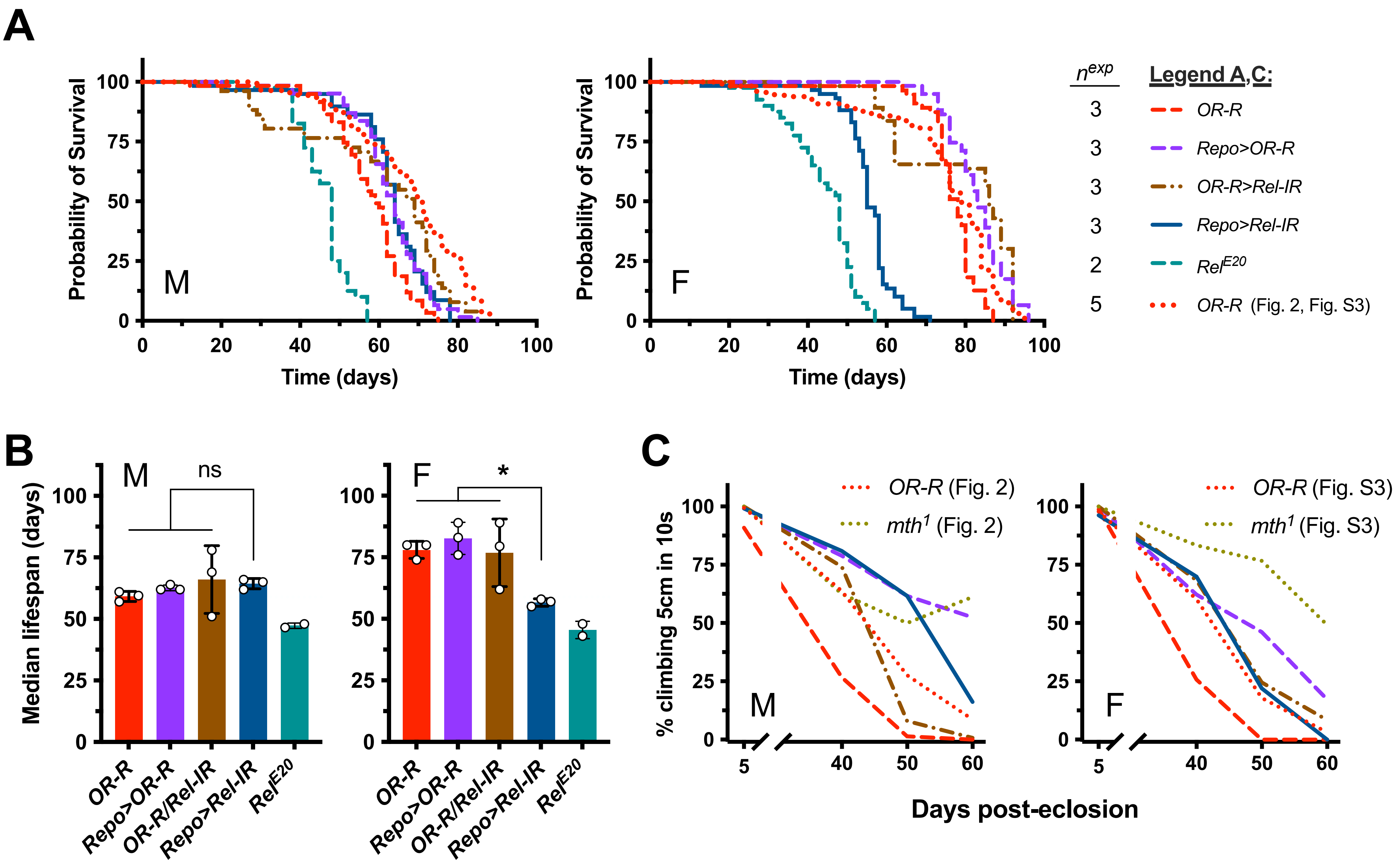


**Fig. S8. Silencing *Relish* in glia does not extend lifespan.** Males (M) and females (F) shown in separate panels. A) Silencing Relish in glia provides no lifespan benefit in males, and was even associated with reduced longevity in females (*Repo>Rel-IR* genotype: *UAS-Rel-IR/+; Repo-Gal4/+*). B) Median lifespans of *Repo* and *Rel-IR* combinations. P < .05 = "*". C) Climbing pass rates of flies with various combinations of *Repo* and *Rel-IR* systematic crosses showing linear progression of climbing competence loss. The *Repo-Gal4* genetic background seems to improve climbing competence into old age independent of the RNAi construct in males, and perhaps slightly in females. Of note, the lifespan and/or climbing of *OR-R* wild-type flies in these experiments was poor compared to the previous *OR-R* experiments shown in Fig. 2. We therefore included the Fig. 2 OR-R lifespan and climbing (and *mth*[1] climbing) in the present figure (A, C) for context, and for ease of direct comparisons.

Disease Models & Mechanisms • Supplementary information

Fig. S9

↑ increases with age ↓ decreases with age ≈ about equal after aging

| Wild-type | Body (sans head) | | | | Head | | | |
|---|---|---|---|---|---|---|---|---|
| | *DptA* | *Drs* | *AttA* | *Dro* | *DptA* | *Drs* | *AttA* | *Dro* |
| ***Oregon-R*** | ↑ | ↑ | ↑↑↑ | ↑↑↑ | ↑ | ↓ | ↓↓↓ | ↓↓↓ |
| ***Exelexis*** | ↑↑ | ≈ | ↑↑ | ↑↑↑ | ↑ | ↑ | ≈ | ↑↑ |
| ***$w^{1118}$ VDRC*** | ↑ | ↓ | ↑ | ≈ | ↑↑ | ↓ | ≈ | ↑↑ |
| ***Canton S*** | ≈ | ≈ | ↑ | ≈ | ≈ | ≈ | ≈ | ↓ |
| Overall trend: | up | no change | up | mixed | up | no change | no change | mixed |

**Fig. S9. No consistent signal of high AMP expression in the head upon aging.** We aged four wild-types (*Oregon-R, Exelexis, $w^{1118}$ VDRC, and Canton S*) for 40 days and compared AMP expression in 40 day old flies to 5 day old flies, specifically in heads or decapitated bodies. While Imd-responsive AMPs were somewhat consistently upregulated in the body with aging, there was little consistent upregulation of AMPs in the head with aging. When present, upregulation was often minor compared to the expression seen during a systemic immune response for those AMP genes (e.g. at 40dpe *DptA* was induced anywhere from 2-50x in the head, or 5-130x in the body, while induction upon infection often reaches 500-1000x using the same normalization procedure). Underlying data and additional qPCR data from a separate experiment following AMP expression in the heads of aging flies at 8, 15, and 30 days are provided in Table S2.

Disease Models & Mechanisms • Supplementary information

**Table S1. Median lifespan and other summary statistics from all genotypes across all experiments, separated by genotype * experiment * sex.** Tabs within this excel file are named according to which experiment they relate to. Full survival raw data are available on FigShare.

Click here to download Table S1

**Table S2. AMP expression data in the head. Contains data related to Fig. S9 for four wild-type flies collected at 5dpe and 40dpe.** Includes additional data on AMP expression in the heads of aged flies at 8, 15, and 30 days post-eclosion.

Click here to download Table S2

**Table S3. Description of fly stocks and qPCR primers used in this study.**

Click here to download Table S3

**File S1. Supplemental text.** This supplementary discussion addresses issues and limitation of our study that we encountered when combining mutations in the DrosDel isogenic background. We discuss the use of median lifespan as our primary readout. There is also discussion of the unique, but minor, climbing effect of *Mtk* and *Drs* mutation in the light of a recent study, but also with the caveat of $Mtk^{R1}$ and $Drs^{R1}$ having a $white^{+}$ transgene insertion that may affect aging and neurodegeneration. Also contains discussion of the consideration of sex*genotype interactions as vial effects, and how future studies may better assess AMP interactions with sex and aging.

Click here to download File S1
